# Design, synthesis and biological activities of echinopsine derivatives containing acylhydrazone moiety

**DOI:** 10.1038/s41598-022-06775-7

**Published:** 2022-02-21

**Authors:** Peipei Cui, Mingjiang Cai, Yanan Meng, Yan Yang, Hongjian Song, Yuxiu Liu, Qingmin Wang

**Affiliations:** 1 College of Arts, Taiyuan University of Technology, Taiyuan, 030024 People’s Republic of China; 2 College of Biomedical Engineering, Taiyuan University of Technology, Taiyuan, 030024 People’s Republic of China; 3 State Key Laboratory of Elemento-Organic Chemistry, College of Chemistry, Frontiers Science Center for New Organic Matter, Collaborative Innovation Center of Chemical Science and Engineering (Tianjin), Nankai University, Tianjin, 300071 People’s Republic of China

**Keywords:** Medicinal chemistry, Organic chemistry, Chemical synthesis

## Abstract

Based on the broad-spectrum biological activities of echinopsine and acylhydrazones, a series of echinopsine derivatives containing acylhydrazone moieties have been designed, synthesized and their biological activities were evaluated for the first time. The bioassay results indicated that most of the compounds showed moderate to good antiviral activities against tobacco mosaic virus (TMV), among which echinopsine (**I**) (inactivation activity, 49.5 ± 4.4%; curative activity, 46.1 ± 1.5%; protection activity, 42.6 ± 2.3%) and its derivatives **1** (inactivation activity, 44.9 ± 4.6%; curative activity, 39.8 ± 2.6%; protection activity, 47.3 ± 4.3%), **3** (inactivation activity, 47.9 ± 0.9%; curative activity, 43.7 ± 3.1%; protection activity, 44.6 ± 3.3%), **7** (inactivation activity, 46.2 ± 1.6%; curative activity, 45.0 ± 3.7%; protection activity, 41.7 ± 0.9%) showed higher anti-TMV activity in vivo at 500 mg/L than commercial ribavirin (inactivation activity, 38.9 ± 1.4%; curative activity, 39.2 ± 1.8%; protection activity, 36.4 ± 3.4%). Some compounds exhibited insecticidal activities against *Plutella xylostella*, *Mythimna separate* and *Spodoptera frugiperda.* Especially, compounds **7** and **27** displayed excellent insecticidal activities against *Plutella xylostell* (mortality 67 ± 6% and 53 ± 6%) even at 0.1 mg/L. Additionally, most echinopsine derivatives exhibited high fungicidal activities against *Physalospora piricola and Sclerotinia sclerotiorum*.

## Introduction

Plant virus diseases can be caused by more than 900 viruses, which reduce grain production and lead to huge economic losses all over the world^1–3^. As a well-studied plant virus, tobacco mosaic virus (TMV) belongs to single-stranded RNA virus of the family togaviridae^4^ and it can infect 268 species of plants in 38 families, such as tobacco, tomato, pepper, cucumber, causing their leaves to grow spots, wither and even leading to yield reduction^5–7^. Although commercially available plant virus inhibitors ningnanmycin and ribavirin are widely used to control TMV, their inhibitory effects are lower than 60%^8^. Thus, the development of efficient alternative TMV inhibitors is still in great request.

Natural products are an important source of plant virus inhibitor discovery. Compared with traditional synthetic plant virus inhibitor, plant virus inhibitor derived from natural products have many advantages, including low toxic, environmentally friendly, easy to decompose and specific to target species, *etc*^9,10^. Song et al*.* reported that the EC_50_ value of purine nucleoside derivative for the inactivating activity against TMV was 48 mg/L, which was better than that of ningnanmycin (88 mg/L)^11^. Li et al*.* first found that phenanthroindolizidine alkaloid, (R)-antofine, exhibited a good inhibitory effect against TMV^12^. Wang et al*.* found some β-carboline analogues^7^, hemigossypol^13^, dehydrobufotenine derivatives^14^, pityriacitrin marine alkaloids^15^, pulmonarin alkaloids^16^ and hamacanthin derivatives^17^ exhibited higher anti-TMV activities than ningnanmycin. Many other natural alkaloids derivatives were also developed as potential TMV inhibitors^18–27^. Although a variety of natural product derivatives have been found to exhibit high anti-TMV activity, few of them have been applied successfully in agriculture. Thus, it is necessary to discover novel natural TMV inhibitors with diverse structures.

**Figure 1:**
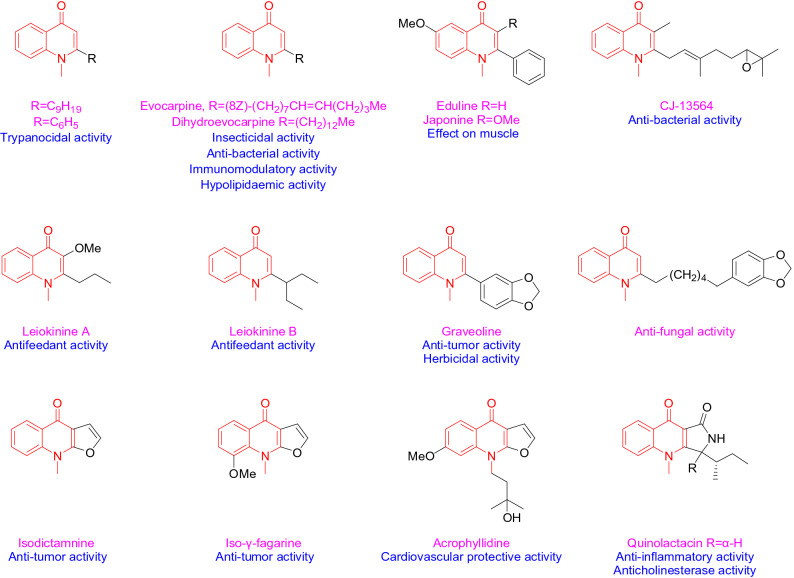
Natural products and drugs containing the core structure of echinopsine.

**Figure 2:**
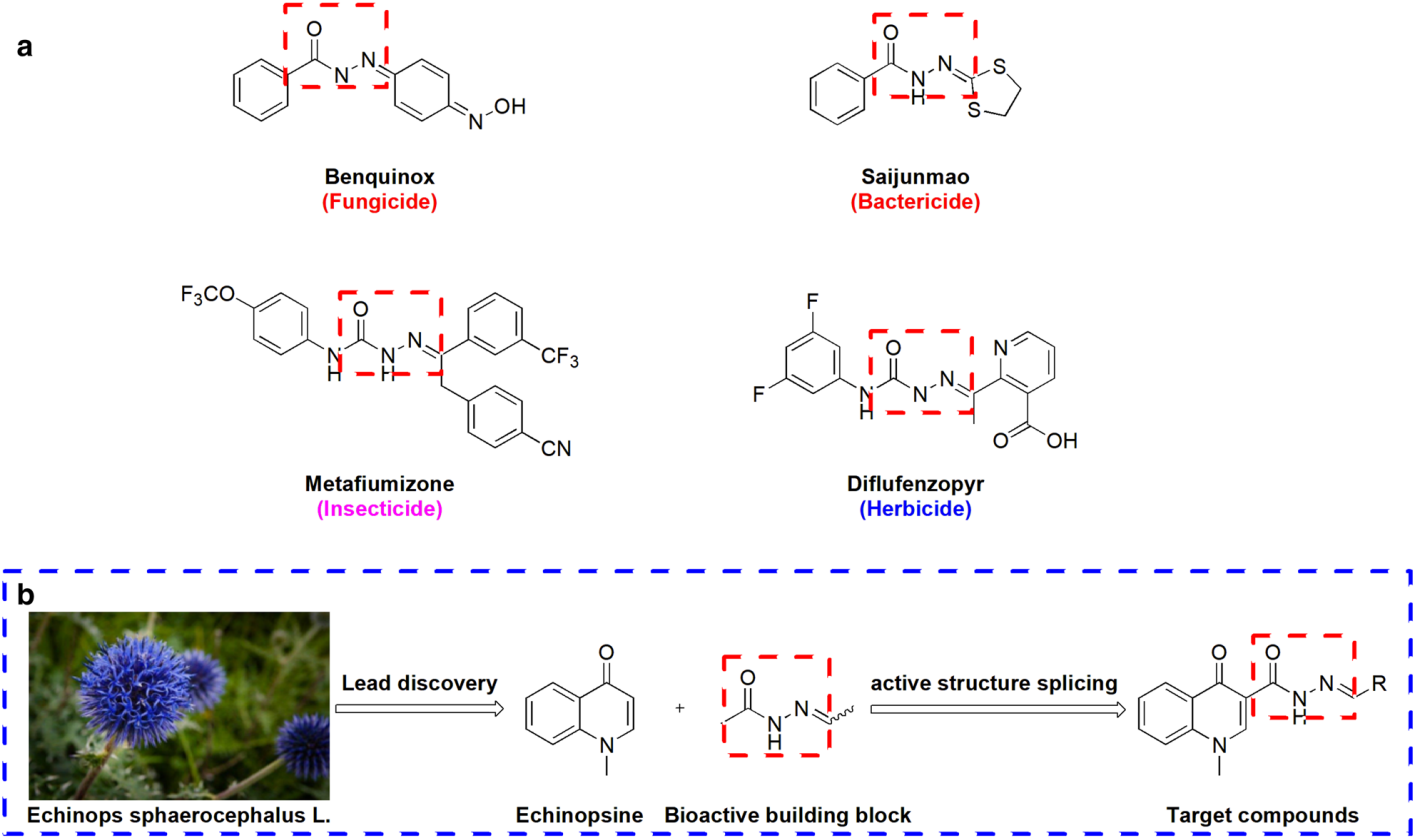
(**a**) Bioactive drugs containing acylhydrazone moieties; (**b**) design strategy for the target molecules.

Echinopsine is a quinoline alkaloid isolated from *Echinops sphaerocephalus *L., the root of which was used as traditional Chinese medicine for treatment of deep-rooted breast carbuncles, ulcer, sodoku and breast milk stoppage. Although the bioactivity of *Echinops sphaerocephalus *L. extract has been widely studied^28^, the biological activity of echinopsine is still not clear. The anti-TMV activity of echinopsine has not been reported so far. However, a variety of natural alkaloids containing echinopsine moiety showed herbicidal, insecticidal, bactericidal, anti-tumor, antifungal and antifeedant activities, etc. (Fig. 1)^29^, indicating echinopsine moiety has potential broad-spectrum biological activities. Based on this, the anti-TMV activity of echinopsine was investigated by our group and the result shows that the inactivation, curative and protection activities of echinopsine (49.5 ± 4.4%, 46.1 ± 1.5% and 42.6 ± 2.3% at 500 mg/L, in Table 1) were higher than that of ribavirin (38.9 ± 1.4%, 39.2 ± 1.8%, 36.4 ± 3.4%, at 500 mg/L). The biological activities of acylhydrazone compounds have always been the focus of pharmacological research^30–33^. Variety of compounds with acylhydrazone functional group (−CONHN=) showed good bactericidal, herbicidal or insecticidal activities, such as benquinox^34^, saijunmao^35^, metaflumizone^36^ and diflufenzopyr^37^. Based on the high biological activities of echinopsine and acylhydrazone structure, in order to find echinopsine derivatives with higher anti-TMV activities and summarize their structure–activity relationship, a series of echinopsine derivatives containing acylhydrazone moieties were designed, synthesized and characterized in this work (Fig. 2). Their anti-TMV activities were studied for the first time. Besides, in order to see if these compounds have broad spectrum bioactivity, their insecticidal and fungicidal activities were also investigated.

**Table 1:** In vivo antiviral activities of compounds** 1**–**27** and echinopsine against TMV.

Compounds	Concentration(mg/L)	Relative inhibition rate (%)		
		Inactivation effect	Curative effect	Protection effect
1	500	44.9 ± 4.6	39.8 ± 2.6	47.3 ± 4.3
2	500	13.7 ± 3.8	–	–
3	500	47.9 ± 0.9	43.7 ± 3.1	44.6 ± 3.3
4	500	35.9 ± 1.4	–	–
5	500	20.6 ± 2.6	–	–
6	500	32.3 ± 1.7	–	–
7	500	46.2 ± 1.6	45.0 ± 3.7	41.7 ± 0.9
8	500	34.1 ± 2.4	–	–
9	500	31.8 ± 2.0	–	–
10	500	30.5 ± 0.3	–	–
11	500	12.0 ± 1.6	–	–
12	500	8.4 ± 4.6	–	–
13	500	42.9 ± 4.4	31.1 ± 2.8	35.8 ± 3.0
14	500	39.7 ± 4.1	–	–
15	500	26.1 ± 2.8	–	–
16	500	28.4 ± 1.8	–	–
17	500	16.0 ± 1.2	–	–
18	500	23.4 ± 3.7	–	–
19	500	12.5 ± 4.8	–	–
20	500	19.3 ± 3.9	–	–
21	500	25.6 ± 3.4	–	–
22	500	40.5 ± 3.5	34.7 ± 4.0	38.3 ± 4.0
23	500	33.2 ± 4.1	–	–
24	500	7.3 ± 2.8	–	–
25	500	4.0 ± 0.5	–	–
26	500	26.4 ± 3.3	–	–
27	500	38.9 ± 2.5	–	–
Echinopsine	500	49.5 ± 4.4	46.1 ± 1.5	42.6 ± 2.3
Ribavirin	500	38.9 ± 1.4	39.2 ± 1.8	36.4 ± 3.4

## Materials and methods

### Instruments

^1^H NMR spectra were obtained at 400 MHz using a Bruker AV400 spectrometer in CDCl_3_ or DMSO-*d*_*6*_ solution with tetramethylsilane as the internal standard. HRMS data were obtained on an FTICR-MS instrument (Ionspec 7.0 T). The melting points were determined on an X-4 binocular microscope melting point apparatus without correction.

### Biological assay

The anti-TMV, insecticidal and fungicidal activities of the synthesized compounds were tested using our previously reported methods^38,39^ and the methods can also be found in the “Supporting Information SI”.

### General synthesis

**Figure 3:**
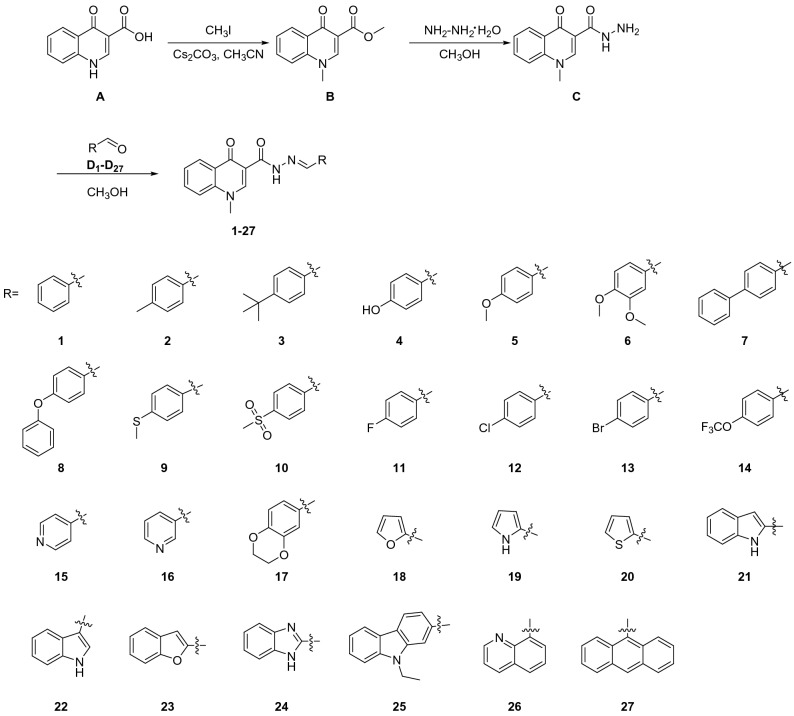
Synthesis of echinopsine acylhydrazone derivatives.

Ribavirin (Topscience Co., Ltd.), chlorothalonil (Bailing Agrochemical Co., Ltd.), carbendazim (Bailing Agrochemical Co., Ltd.) and other reagents were purchased from commercial sources and used as received. All anhydrous solvents were dried and purified according to standard techniques. The synthetic routes were given in Fig. 3.

Echinopsine was prepared according to literature^40^.

### Synthesis of methyl 1-methyl-4-oxo-1,4-dihydroquinoline-3-carboxylate (**B**)

To a round bottomed flask (500 mL) were added compound **A** (1.89 g, 10 mmol), Cs_2_CO_3_ (1.89 g, 10 mmol) and acetonitrile (300 mL). The reaction suspension was stirred for half an hour at room temperature and methyl iodide (4.24 g, 30 mmol) was added. The mixture was refluxed for 6 h. Water (200 mL) was added and the reaction mixture was extracted with ethyl acetate for three times. The organic phases were combined, washed with brine, dried over anhydrous Na_2_SO_4_ and evaporated under reduced pressure. The residue was subjected to column chromatography eluted with dichloromethane / methanol (v/v, 50/1) to give compound **B** as a white solid (1.98 g, 91.2% yield); mp 189–190 °C. ^1^H NMR (400 MHz, CDCl_3_) *δ* 8.53 (d, *J* = 8.0 Hz, 1H), 8.50 (s, 1H), 7.71 (dd, *J* = 8.0, 8.0 Hz, 1H), 7.48–7.42 (m, 2H), 3.93 (s, 3H), 3.89 (s, 3H); ^13^C NMR (100 MHz, CDCl_3_) *δ* 174.5, 166.7, 150.1, 139.9, 132.9, 129.1, 128.0, 125.5, 115.7, 110.7, 52.3, 41.5; HRMS (ESI) calcd. for C_12_H_12_NO_3_ [M+H]^+^ 218.0812, found 218.0811.

### Synthesis of 1-methyl-4-oxo-1,4-dihydroquinoline-3-carbohydrazide (**C**)

Compound **B** (4.34 g, 20 mmol) and hydrazine hydrate (12.50 g, 200 mmol, 80%) were dissolved in methanol (300 mL). The mixture was refluxed for 8 h. The mixture was concentrated under reduced pressure until a large amount of solid precipitated. The mixture was filtered, washed with a small amount of methanol to give compound **C** as a white solid (4.20 g, 96.8% yield); mp 273–275 °C. ^1^H NMR (400 MHz, DMSO*-d*_6_) *δ* 10.67 (s, 1H), 8.86 (s, 1H), 8.34 (d, *J* = 8.0 Hz, 1H), 7.88–7.81 (m, 2H), 7.55 (dd, *J* = 8.0, 8.0 Hz, 1H), 4.58 (d, *J* = 4.4 Hz, 2H), 4.02 (s, 3H); ^13^C NMR (100 MHz, DMSO*-d*_6_) *δ* 175.1, 163.9, 148.3, 139.8, 133.0, 126.8, 126.0, 125.1, 117.5, 110.2, 41.2; HRMS (ESI) calcd. for C_11_H_12_N_3_O_2_ [M+H]^+^ 218.0924, found 218.0920.

### General procedure for the preparation of compounds **1**–**27**

To a round bottomed flask (100 mL) were added methanol (50 mL), compound **C** (3 mmol), one benzaldehyde from **D**_**1**_–**D**_**27**_ (3 mmol) and *p-*methylbenzene sulfonic acid (0.6 mmol). The reaction suspension was refluxed for 8 h. The reaction suspension was cooled to room temperature and partial methanol was evaporated under reduced pressure until a large amount of precipitation precipitated. The precipitate was filtered and washed several times with cool methanol to afford compounds **1**–**27**. Data for compounds **1**–**27** can be found in the “Supporting Information SI”.

## Results and discussion

### Synthesis

The preparation of compound **B** was carried out according to literature^41^ (Fig. 3). Acetonitrile was used as solvent instead of DMF and the reaction was accomplished in 91.2% yield. Then product **B** reacted with hydrazine hydrate under reflux to afford hydrazine **C**, which can react subsequently with aldehyde **D**_**1**_–**D**_**27**_ to give hydrazine **1**–**27** as products in 52.7–95.3% yields. During the synthesis of acylhydrazone **1**–**27**, only trans isomers were obtained, which may due to the fact that trans isomers are more stable than cis isomers thermodynamically. Compounds **1**–**27** can precipitate from methanol, which made the purification of acylhydrazone derivatives easy and suitable for large-scale production.

### In vivo anti-TMV activity

The results of anti-TMV activities in vivo (inactivation, curative, and protection mode) of echinopsine and compounds **1**–**27** are listed in Table 1. In order to make the antiviral activity results more reliable, commercial plant virus inhibitor ribavirin was taken as control. In our previous work, the highly antiviral lead echinopsine was found, based on which a series of echinopsine derivatives containing acylhydrazone structure were synthesized in this work to study the influence of the variation of the functional groups on the antiviral activities of echinopsine. The antiviral results (Table 1) showed that some echinopsine acylhydrazone compounds exhibited moderate to good anti-TMV activity compared with ribavirin. Especially, the inactivation activity, curative activity, protection activity of compounds **1** (44.9 ± 4.6, 39.8 ± 2.6 and 47.3 ± 4.3%, 500 mg/L), 3 (47.9 ± 0.9, 43.7 ± 3.1, and 44.6 ± 3.3%, 500 mg/L), 7 (46.2 ± 1.6, 45.0 ± 3.7, and 41.7 ± 0.9%, 500 mg/L) were obviouly higher than that of commercialized anti-plant virus agent ribavirin (38.9 ± 1.4, 39.2 ± 1.8, and 36.4 ± 3.4%, 500 mg/L).

For derivatives containing substituted phenyl (**1**–**14**), the electronic effect of the substituents on phenyl has an effect on the anti-TMV activities. The introduction of electron-withdrawing and electron-donating substituents led to the decrease of anti-TMV activities. For example, the structure–activity relationship shows the following: non-substituent (**1**) > *p*-hydroxyl (**4**) > *p-*phenoxy (**8**) > *p*-methylthio (**9**) > *p*-methoxy (**5**), non-substituent (**1**) > *p*-bromo substituent (**13**) > *p*-methylsulfonyl (**10**) > *p*-fluorosubstituent (**11**) > *p*-chloro substituent (**12**). However, there is no obvious linear relationship between anti-TMV activity and electron-donating and electron-withdrawing ability. For example, the structure–activity relationship shows the following: p-bromo substituent (**13**) > *p*-trifluoromethoxy substituent (**14**) > *p*-fluoro substituent (**11**) > *p*-chloro substituent (**12**), while the activity of compound **13** at 500 mg/L (inactivation activity, 42.9 ± 4.4%; curative activity, 31.1 ± 2.8%; protection activity, 35.8 ± 3.0%) is equivalent to that of ribavirin. The size of substituents also has an effect on the activities. For example, the activities of derivatives with a *p*-tert-butyl (**3**) and *p*-phenyl substituent (**7**) are higher than that with no substituents (**1**). Mono substitution or multi substitution on the benzene ring affected anti-TMV activity to a certain extent, for instance, compared with compounds **5** (inactivation, 20.6 ± 2.6%, 500 mg/L), the disubstituted compound **6** (inactivation, 32.3 ± 1.7%, 500 mg/L) exhibited higher activity.

The anti-TMV activities of compounds **15**–**26** containing heterocyclic ring reduced obviously compared with that of compounds containing benzene ring (**1**). Compound **22**, showed the highest activities at 500 mg/L (inactivation activity, 40.5 ± 3.5%; curative activity, 34.7 ± 4.0%; protection activity, 38.3 ± 4.0%), which was equivalent to that of ribavirin. However, the activity was greatly reduced when the benzene ring was changed to an anthracene ring, that is, the activities of compound **27** (inactivation, 38.9 ± 2.5%, 500 mg/L) was lower than that of compound **1 **(inactivation, 44.9 ± 4.6%, 500 mg/L).

Compound **3** showed the highest activities at 500 mg/L (inactivation activity, 47.9 ± 0.9%; curative activity, 43.7 ± 3.1%; protection activity, 44.6 ± 3.3%), which is significantly higher than that of ribavirin. Thus, this compound (**3**) can be selected as an anti-TMV candidate drug for further study.

### Insecticidal activities

The insecticidal activities of the target compounds **1**–**27** and echinopsine against Lepidoptera pests, such as diamondback moth (*Plutella xylostella*), cotton bollworm (*Helicoverpa armigera*), corn borer (Ostrinia nubilalis), oriental armyworm (*Mythimna separata*) and fall armyworm (*Spodoptera frugiperda* (J. E. Smith)) are listed in Tables 2 and 3, echinopsine was taken as control.

**Table 2:** Insecticidal activity of compounds **1**–**27** and echinopsine against Diamond Back Moth (*Plutella xylostella*).

Compounds	Larvicidal activity (mortality %) at concn (mg/L)					
	600	200	100	10	1	0.1
1	73 ± 6	–	–	–	–	–
2	0	–	–	–	–	–
3	0	–	–	–	–	–
4	40 ± 10	–	–	–	–	–
5	53 ± 6	–	–	–	–	–
6	70 ± 0	–	–	–	–	–
7	100 ± 0	100 ± 0	100 ± 0	100 ± 0	90 ± 0	67 ± 6
8	0	–	–	–	–	–
9	80 ± 0	57 ± 6	–	–	–	–
10	77 ± 0	–	–	–	–	–
11	0	–	–	–	–	–
12	67 ± 6	–	–	–	–	–
13	60 ± 10	–	–	–	–	–
14	100 ± 0	90 ± 0	77 ± 6	–	–	–
15	100 ± 0	100 ± 0	90 ± 0	60 ± 0	–	–
16	67 ± 6	–	–	–	–	–
17	40 ± 10	–	–	–	–	–
18	70 ± 0	–	–	–	–	–
19	83 ± 6	70 ± 0	–	–	–	–
20	0	–	–	–	–	–
21	100 ± 0	100 ± 0	100 ± 0	90 ± 0	57 ± 6	–
22	0	–	–	–	–	–
23	100 ± 0	90 ± 0	73 ± 6	47 ± 6	–	–
24	83 ± 6	60 ± 0	–	–	–	–
25	100 ± 0	93 ± 6	80 ± 0	60 ± 0	–	–
26	100 ± 0	100 ± 0	80 ± 0	60 ± 0	–	–
27	100 ± 0	100 ± 0	100 ± 0	100 ± 0	80 ± 0	53 ± 6
Echinopsine	90 ± 0	70 ± 0	–	–	–	–

**Table 3:** Insecticidal activity of compounds** 1**–**27** and echinopsineagainst Cotton Bollworm (*Helicoverpa armigera*), Corn Borer (*Ostrinia nubilalis*), Oriental Armyworm (*Mythimna separata*), Fall Armyworm (*Spodoptera Frugiperda* (J. E. Smith)).

Compounds	600 mg/L, mortality/%			
	*H. armigera*	*O. nubilalis*	*M. separata*	*S. frugiperda*
1	27 ± 6	0	50 ± 0	47 ± 6
2	20 ± 0	10 ± 0	60 ± 0	30 ± 0
3	10 ± 0	0	70 ± 0	70 ± 0
4	10 ± 0	7 ± 6	40 ± 0	50 ± 0
5	0	0	100 ± 0/100 ± 0^a^/40 ± 0^b^	100 ± 0/100 ± 0^a^/17 ± 6^b^
6	0	0	10 ± 0	20 ± 0
7	30 ± 0	20 ± 0	100 ± 0/30 ± 0^a^	47 ± 6
8	17 ± 6	7 ± 6	50 ± 0	47 ± 6
9	37 ± 6	20 ± 0	100 ± 0/100 ± 0^a^/60 ± 0^b^/20 ± 0^c^	60 ± 0
10	10 ± 0	0	100 ± 0/60 ± 0^a^	30 ± 0
11	17 ± 6	0	40 ± 0	47 ± 6
12	10 ± 0	13 ± 6	30 ± 0	30 ± 0
13	23 ± 6	10 ± 0	60 ± 0	37 ± 6
14	7 ± 6	0	100 ± 0/100 ± 0^a^/40 ± 0^b^	40 ± 0
15	10 ± 0	0	40 ± 0	10 ± 0
16	30 ± 0	20 ± 0	100 ± 0/60 ± 0^a^	30 ± 0
17	30 ± 0	20 ± 0	70 ± 0	57 ± 6
18	27 ± 6	0	60 ± 0	23 ± 6
19	33 ± 6	0	80 ± 0	70 ± 0
20	7 ± 6	0	50 ± 0	50 ± 0
21	0	0	100 ± 0/100 ± 0^a^/37 ± 6^b^	100 ± 0/100 ± 0^a^/10 ± 0^b^
22	10 ± 0	0	20 ± 0	20 ± 0
23	47 ± 6	37 ± 6	100 ± 0/50 ± 0^a^	67 ± 6
24	23 ± 6	10 ± 0	100 ± 0/100 ± 0^a^/100 ± 0^b^/30 ± 0^c^	100 ± 0/100 ± 0^a^/100 ± 0^b^/17 ± 6^c^
25	37 ± 6	7 ± 6	100 ± 0/100 ± 0^a^/27 ± 6^b^	100 ± 0/100 ± 0^a^/10 ± 0^b^
26	17 ± 6	0	50 ± 0	10 ± 0
27	27 ± 6	7 ± 6	30 ± 0	10 ± 0
Echinopsine	30 ± 0	20 ± 0	100 ± 0/70 ± 0^a^	50 ± 0

^a^Mortality at 200 mg/L, ^b^Mortality at100 mg/L, ^c^Mortality at 50 mg/L.

The result showed that echinopsine and some derivatives showed broad spectrum insecticidal activities. Most of the compounds exhibited moderate to good larvicidal activities against *P. xylostella*. For derivatives containing substituted phenyl (**1**–**14**) and anthranyl (**27**), compounds **7**, **14** and **27** exhibited 100 ± 0% mortality at 600 mg/L. In particular, compounds **7** and **27** still showed 67 ± 6% and 53 ± 6% mortality even at 0.1 mg/L. Compounds **15**, **21**, **23**, **25** and **26** containing heterocyclic ring also showed 100 ± 0% mortality at 600 mg/L, which was better than echinopsine (90 ± 0% at 600 mg/L) (Table 2).

At the same time, the insecticidal activities of compounds **15**–**26** containing heterocyclic ring against *M. separata* and *S. frugiperda* were higher than that of compounds **1**–**14** containing benzene ring. The compounds **5**, **9**, **14**, **21**, **24 **and **25** exhibited higher activities (100 ± 0% at 200 mg/L) against *M. separata* than that of echinopsine (70 ± 0% at 200 mg/L). Especially, compounds **9** and **24** showed 20 ± 0% and 30 ± 0% mortality at 50 mg/L. In addition, the compounds **5**, **21**, **24**, and **25** showed much higher activities (100 ± 0% at 200 mg/L) against *S. frugiperda* than that of echinopsine (50 ± 0% at 600 mg/L). Especially, compounds **24** still showed 17 ± 6% mortality at 50 mg/L (Table 3).

### Fungicidal activity

The fungicidal results of compounds **1**–**27** and echinopsine are listed in Table 4. The commercial fungicide carbendazim and chlorothalonil were used as positive control. Overall, echinopsine and their derivatives exhibited broad-spectrum fungicidal activities against 14 kinds of phytopathogenic fungi. Most compounds showed relatively high fungicidal activities for *Physalospora piricola and Sclerotinia sclerotiorum*, among which the fungicidal activities of compounds **1**–**14** containing substituted phenyl were relatively higher than compounds **15**–**26** containing heterocyclic rings. Compound **13** and **14** showed more than 50% inhibitory rate against five and six fungi respectively. Compound **2** showed the widest spectrum of fungicidal activity, with more than 60% inhibitory rate against eight fungi. Compound **7** exhibits 89.0 ± 1.9% inhibitory rate against *Rhizoctonia cerealis* at 50 mg/L, higher than carbendazim and chlorothalonil.

**Table 4:** Fungicidal activity of compounds** 1**–**27** and echinopsine against fourteen kinds of phytopathogens (50 mg/L, inhibition rate/%).

Compounds	Fc^a^	Ch	Pp	As	Fg	Fm	Ss	Pc	Rc	Bm	Wa	Rs	Bc	Mg
1	12.3 ± 1.2	6.7 ± 0.9	50.0 ± 1.3	38.9 ± 0.6	35.5 ± 2.3	11.6 ± 1.7	66.7 ± 2.0	19.4 ± 1.2	14.6 ± 0.8	9.6 ± 1.3	10.5 ± 2.4	27.8 ± 1.9	23.8 ± 0.7	11.1 ± 1.4
2	38.6 ± 1.8	76.7 ± 1.6	66.1 ± 1.9	61.1 ± 0.9	41.9 ± 1.3	65.1 ± 0.8	69.4 ± 1.4	22.6 ± 0.9	64.6 ± 1.9	53.8 ± 2.1	52.6 ± 2.8	63.9 ± 1.8	38.1 ± 2.0	88.9 ± 1.7
3	36.8 ± 1.8	6.7 ± 0.8	66.1 ± 1.7	38.9 ± 2.7	25.8 ± 0.9	25.6 ± 1.9	77.8 ± 2.3	16.1 ± 0.7	62.2 ± 0.8	7.7 ± 1.3	12.3 ± 1.7	5.6 ± 0.8	38.1 ± 1.4	5.6 ± 1.1
4	19.3 ± 0.8	13.3 ± 1.4	35.5 ± 0.9	50.0 ± 1.2	41.9 ± 0.6	27.9 ± 0.7	75.0 ± 1.8	16.1 ± 1.7	13.4 ± 0.8	11.5 ± 2.3	17.5 ± 1.5	36.1 ± 2.2	23.8 ± 1.9	77.8 ± 1.6
5	17.5 ± 0.7	10.0 ± 1.7	66.1 ± 2.3	16.7 ± 1.4	22.6 ± 1.9	34.9 ± 2.4	69.4 ± 1.7	19.4 ± 0.9	13.4 ± 1.1	5.8 ± 1.2	15.8 ± 1.8	27.8 ± 0.8	33.3 ± 2.3	22.2 ± 0.7
6	45.6 ± 1.8	13.3 ± 3.3	82.3 ± 2.4	38.9 ± 0.9	25.8 ± 1.2	16.3 ± 1.0	80.6 ± 1.9	22.6 ± 2.2	25.6 ± 0.7	11.5 ± 1.9	19.3 ± 0.9	11.1 ± 2.1	33.3 ± 0.9	5.6 ± 1.3
7	36.8 ± 0.6	10.0 ± 0.7	66.1 ± 1.3	38.9 ± 0.8	16.1 ± 0.9	25.6 ± 1.4	66.7 ± 1.2	9.7 ± 1.1	89.0 ± 1.9	7.7 ± 0.7	12.3 ± 1.3	36.1 ± 0.8	19.0 ± 1.1	44.4 ± 1.7
8	36.8 ± 0.6	10.0 ± 1.9	74.2 ± 2.2	38.9 ± 1.8	22.6 ± 1.4	27.9 ± 1.9	69.4 ± 0.7	22.6 ± 0.8	53.7 ± 1.2	15.4 ± 0.8	17.5 ± 1.4	11.1 ± 0.8	57.1 ± 2.3	5.6 ± 0.5
9	8.8 ± 1.3	33.3 ± 1.4	88.7 ± 0.6	38.9 ± 1.2	19.4 ± 1.8	39.5 ± 0.9	77.8 ± 2.3	16.1 ± 2.4	12.2 ± 0.6	11.5 ± 1.1	12.3 ± 0.7	5.6 ± 1.4	19.0 ± 2.7	11.1 ± 0.8
10	45.6 ± 1.9	46.7 ± 2.3	82.3 ± 1.5	33.3 ± 1.2	25.8 ± 2.8	46.5 ± 2.4	77.8 ± 1.3	16.1 ± 1.7	56.1 ± 1.3	15.4 ± 1.8	21.1 ± 1.1	13.9 ± 0.6	23.8 ± 0.9	22.2 ± 1.7
11	15.8 ± 0.6	6.7 ± 1.1	50.0 ± 2.4	44.4 ± 3.1	19.4 ± 0.7	34.9 ± 1.2	61.1 ± 0.9	6.5 ± 0.8	11.0 ± 0.3	7.7 ± 0.6	15.8 ± 1.6	11.1 ± 0.6	23.8 ± 1.4	11.1 ± 0.7
12	22.8 ± 1.8	53.3 ± 2.2	51.6 ± 3.4	16.7 ± 2.3	25.8 ± 0.8	55.8 ± 1.6	63.9 ± 2.9	6.5 ± 1.0	35.4 ± 3.1	34.6 ± 1.7	26.3 ± 1.8	5.6 ± 0.7	23.8 ± 1.5	11.1 ± 1.1
13	36.8 ± 2.0	33.3 ± 1.1	87.1 ± 2.3	50.0 ± 2.8	29.0 ± 2.2	37.2 ± 1.4	61.1 ± 0.8	9.7 ± 0.9	74.4 ± 1.4	48.1 ± 2.8	15.8 ± 0.7	38.9 ± 1.8	23.8 ± 2.3	66.7 ± 1.9
14	19.3 ± 3.3	60.0 ± 1.9	74.2 ± 2.2	27.8 ± 1.2	6.5 ± 1.4	65.1 ± 0.8	55.6 ± 3.3	6.5 ± 1.2	50.0 ± 0.9	50.0 ± 2.0	43.9 ± 1.9	8.3 ± 0.9	9.5 ± 1.1	5.6 ± 0.6
15	10.5 ± 1.2	43.3 ± 2.0	51.6 ± 0.7	38.9 ± 0.8	29.0 ± 1.5	18.6 ± 1.7	75.0 ± 0.9	25.8 ± 2.1	13.4 ± 1.4	15.4 ± 0.7	19.3 ± 3.3	19.4 ± 1.2	23.8 ± 0.6	11.1 ± 0.5
16	15.8 ± 0.9	10.0 ± 3.2	33.9 ± 1.2	16.7 ± 1.1	3.2 ± 0.8	25.6 ± 1.3	66.7 ± 2.1	16.1 ± 0.7	37.8 ± 3.1	11.5 ± 0.3	17.5 ± 0.8	11.1 ± 1.9	23.8 ± 1.4	5.6 ± 0.6
17	10.5 ± 0.8	3.3 ± 0.6	74.2 ± 1.9	38.9 ± 2.3	29.0 ± 0.3	20.9 ± 1.2	72.2 ± 1.7	6.5 ± 1.0	17.1 ± 0.9	1.9 ± 1.1	14.0 ± 0.7	25.0 ± 0.9	14.3 ± 0.6	66.7 ± 1.4
18	10.5 ± 0.4	26.7 ± 2.1	71.0 ± 2.9	27.8 ± 1.2	22.6 ± 2.4	39.5 ± 1.7	75.0 ± 3.4	22.6 ± 0.8	39.0 ± 2.2	21.2 ± 1.8	28.1 ± 1.7	30.6 ± 2.4	28.6 ± 2.3	22.2 ± 1.6
19	10.5 ± 1.6	23.3 ± 2.0	71.0 ± 1.8	33.3 ± 1.3	32.3 ± 0.9	20.9 ± 2.2	77.8 ± 1.9	16.1 ± 2.0	19.5 ± 1.6	11.5 ± 1.2	10.5 ± 0.5	50.0 ± 1.5	33.3 ± 1.4	55.6 ± 2.8
20	15.8 ± 0.7	13.3 ± 0.9	41.9 ± 1.2	38.9 ± 3.3	29.0 ± 0.9	0.0 ± 0.0	55.6 ± 1.4	29.0 ± 1.2	13.4 ± 1.9	11.5 ± 0.8	15.8 ± 0.9	13.9 ± 1.3	9.5 ± 1.1	11.1 ± 0.6
21	17.5 ± 1.6	3.3 ± 0.9	40.3 ± 2.2	33.3 ± 1.3	22.6 ± 0.6	23.3 ± 0.7	80.6 ± 2.4	12.9 ± 1.3	14.6 ± 0.8	3.8 ± 1.1	12.3 ± 0.8	27.8 ± 2.2	19.0 ± 1.4	55.6 ± 1.8
22	22.8 ± 1.7	3.3 ± 0.5	50.0 ± 1.2	38.9 ± 1.3	16.1 ± 0.8	20.9 ± 1.2	75.0 ± 1.4	16.1 ± 2.3	63.4 ± 2.0	9.6 ± 0.5	10.5 ± 0.7	13.9 ± 0.9	38.1 ± 1.6	11.1 ± 0.5
23	19.3 ± 0.7	53.3 ± 2.2	45.2 ± 1.5	22.2 ± 1.8	16.1 ± 1.2	48.8 ± 2.1	55.6 ± 1.9	22.6 ± 1.3	56.1 ± 3.2	17.3 ± 0.7	26.3 ± 1.7	19.4 ± 0.7	23.8 ± 2.3	5.6 ± 0.7
24	12.3 ± 0.5	6.7 ± 0.7	35.5 ± 1.2	38.9 ± 2.3	32.3 ± 1.6	16.3 ± 2.1	66.7 ± 1.7	16.1 ± 1.9	26.8 ± 0.9	7.7 ± 0.6	10.5 ± 0.9	22.2 ± 1.4	23.8 ± 0.9	5.6 ± 0.6
25	10.5 ± 2.2	6.7 ± 0.9	50.0 ± 1.4	38.9 ± 2.3	25.8 ± 1.3	34.9 ± 0.8	66.7 ± 0.9	9.7 ± 1.2	17.1 ± 1.8	11.5 ± 0.6	15.8 ± 2.4	5.6 ± 0.6	23.8 ± 2.3	11.1 ± 0.8
26	10.5 ± 0.5	10.0 ± 2.3	87.1 ± 2.4	33.3 ± 1.4	25.8 ± 1.9	25.6 ± 2.2	63.9 ± 3.4	6.5 ± 0.6	11.0 ± 0.8	9.6 ± 1.9	12.3 ± 1.4	5.6 ± 0.9	23.8 ± 1.6	11.1 ± 0.7
27	10.5 ± 2.1	3.3 ± 0.6	74.2 ± 1.6	33.3 ± 2.3	38.7 ± 0.9	16.3 ± 1.1	13.9 ± 1.6	9.7 ± 1.4	18.3 ± 1.8	9.6 ± 0.7	17.5 ± 2.2	5.6 ± 0.8	9.5 ± 0.6	11.1 ± 1.0
Echinopsine	10.5 ± 1.4	20.0 ± 1.9	58.1 ± 2.2	16.7 ± 2.4	19.4 ± 2.2	37.2 ± 0.9	69.4 ± 1.7	16.1 ± 0.8	18.3 ± 2.2	15.4 ± 0.8	14.0 ± 1.3	22.2 ± 1.7	28.6 ± 0.6	11.1 ± 1.2
carbendazim	< 50.0	< 50.0	< 50.0	< 50.0	100.0 ± 0.0	100.0 ± 0.0	100.0 ± 0.0	100.0 ± 0.0	< 50.0	100.0 ± 0.0	< 50.0	100.0 ± 0.0	< 50.0	100.0 ± 0.0
chlorothalonil	100.0 ± 0.0	73.3 ± 1.2	100.0 ± 0.0	100.0 ± 0.0	100.0 ± 0.0	< 50.0	86.4 ± 1.3	91.3 ± 0.9	73.3 ± 0.9	< 50.0	100.0 ± 0.0	100.0 ± 0.0	100.0 ± 0.0	91.3 ± 0.8

^a^*Fc, Fusarium oxysporium f. *sp. *cucumeris; Ch, Cercospora arachidicola Hori; Pp, Physalospora piricola; As, Alternaria solani; Fg, Fusarium graminearum; Fm, Fusarium moniliforme; Ss, Sclerotinia sclerotiorum; Pc, Phytophthora capsici; Rc, Rhizoctonia cerealis; Bm,Bipolaris maydis; Wa, watermelon anthracnose; Rs, Rhizoctonia solani; Bc, Botrytis cinereaand; Mg, magnaporthe grisea*.

In summary, a series of novel echinopsine derivatives containing acylhydrazone moieties were designed, synthesized and their antiviral, insecticidal, and fungicidal activities were studied. The bioassays results showed that most compounds exhibited moderate to good anti-TMV activities in vivo, among which echinopsine (I) and its derivatives 1, 3, 7 showed higher anti-TMV activities than those of ribavirin, which can be used as lead structures for the development of anti-TMV drugs. Some compounds exhibited moderate to good insecticidal activity to *P. xylostella*, *M. separata* and *S.* *frugiperda*. In addition, most of these compounds exhibited good fungicidal activities against *P. piricola* and *S. sclerotiorum*. Further investigation on structural optimization and the mechanism of action are in progress in our laboratory.

## Supplementary Information

Supplementary Information.
