# Supplementary material for: Design, synthesis and biological activities of echinopsine derivatives containing acylhydrazone moiety
Source: Sci Rep. 2022 Feb 21;12:2935. doi: 10.1038/s41598-022-06775-7 (PMC8861054; doi:10.1038/s41598-022-06775-7)
Supplement: Supplementary file 1 — Supplementary Information. [file 41598_2022_6775_MOESM1_ESM.docx]

*Supporting Information*

**Design, Synthesis and Biological Activities of Echinopsine Derivatives Containing Acylhydrazone Moiety**

**Peipei Cui^1^, Mingjiang Cai^2^, Yanan Meng^2^, Yan Yang^2^,* Hongjian Song^3^, Yuxiu Liu^3^, Qingmin Wang^3^***

*^1^College of Arts, Taiyuan University of Technology, Taiyuan 030024, People’s Republic of China*

*^2^College of Biomedical Engineering, Taiyuan University of Technology, Taiyuan 030024, People’s Republic of China*

*^3^State Key Laboratory of Elemento-Organic Chemistry, College of Chemistry, Frontiers Science Center for New Organic Matter, Nankai University, Tianjin 300071, People’s Republic of China*

* Corresponding author: [yy529222@163.com](mailto:yxlwell@163.com) (Y. Yang), [wangqm@nankai.edu.cn](mailto:wangqm@nankai.edu.cn) (Q. -M. Wang).

Data for compounds **1-27**……………………………………………..……………………...S2-S11

^1^H NMR and ^13^C NMR spectrum for echinopsine and compounds **1-27**……………….….S12–S39

Biological assay…………………………………………………………………….....……S40–S43

*Data for* (E)-N'-benzylidene-1-methyl-4-oxo-1,4-dihydroquinoline-3-carbohydrazide **(1)***.* White solid; yield: 95.3%; mp 259 - 260 ^o^C. ^1^H NMR (400 MHz, DMSO*-d_6_*) *δ* 13.30 (s, 1H), 8.99 (s, 1H), 8.43 (s, 1H), 8.39 (d, *J* = 8.0 Hz, 1H), 7.92 - 7.86(m, 2H), 7.77 (d, *J* = 6.4 Hz, 2H), 7.63 - 7.60 (m, 1H), 7.48 - 7.43 (m, 3H), 4.08 (s, 3H); ^13^C NMR (100 MHz, DMSO*-d*_6_) *δ* 175.4, 161.1, 149.4, 147.7, 139.9, 134.4, 133.3, 130.0, 128.8, 127.2, 126.7, 126.0, 125.5, 117.8, 109.8, 41.4; HRMS (ESI) calcd. for C_18_H_16_N_3_O_2_ [M+H]^+^ 306.1237, found 306.1232.

*Data for* (E)-1-methyl-N'-(4-methylbenzylidene)-4-oxo-1,4-dihydroquinoline-3-carbohydrazide **(2)**. White solid; yield: 83.6%; mp 297 - 298 ^o^C. ^1^H NMR (400 MHz, DMSO*-d_6_*) *δ* 13.21 (s, 1H), 8.97 (s, 1H), 8.40 - 8.38 (m, 2H), 7.91 - 7.85 (m, 2H), 7.66 (d, *J* = 7.6 Hz, 2H), 7.62 - 7.59 (m,1H), 7.27 (d, *J* = 7.6 Hz, 2H), 4.07 (s, 3H), 2.35 (s, 3H); ^13^C NMR (100 MHz, DMSO*-d*_6_) *δ* 175.4, 161.0, 149.4, 147.7, 139.9, 139.8, 133.3, 131.7, 129.4, 127.2, 126.7, 126.0, 125.5, 117.7, 109.9, 41.4, 21.1; HRMS (ESI) calcd. for C_19_H_18_N_3_O_2_ [M+H]^+^ 320.1394, found 320.1390.

*Data for* (E)-N'-(4-(tert-butyl)benzylidene)-1-methyl-4-oxo-1,4-dihydroquinoline-3-carbohydrazide **(3)**. White solid; yield: 66.5%; mp 298 - 299 ^o^C. ^1^H NMR (400 MHz, CDCl_3_) *δ* 13.19 (s, 1H), 8.90 (s, 1H), 8.55 (d, *J* = 8.4 Hz, 1H), 8.21 (s, 1H), 7.82 - 7.78 (m, 1H), 7.75 (d, *J* = 8.4 Hz, 2H), 7.58 - 7.54 (m, 2H), 7.42 (d, *J* = 8.4 Hz, 2H), 4.02 (s, 3H), 1.34 (s, 9H); ^13^C NMR (100 MHz, CDCl_3_) *δ* 176.6, 161.8, 153.8, 149.0, 148.6, 140.0, 133.4, 131.4, 127.8, 127.7, 127.4, 125.8, 125.7, 116.1, 111.4, 41.8, 35.0, 31.3; HRMS (ESI) calcd. for C_22_H_24_N_3_O_2_ [M+H]^+^ 362.1863, found 362.1860.

*Data for* (E)-N'-(4-hydroxybenzylidene)-1-methyl-4-oxo-1,4-dihydroquinoline-3-carbohydrazide **(4)**. Yellow solid; yield: 87.2%; mp > 300 °C. ^1^H NMR (400 MHz, DMSO*-d_6_*) *δ* 13.14 (s, 1H), 9.94 (s, 1H), 8.97 (s, 1H), 8.38 (d, *J* = 8.0 Hz, 1H), 8.29 (s, 1H), 7.92 - 7.85 (m, 2H), 7.60 (d, *J* = 8.4 Hz, 3H), 6.84 (d, *J* = 8.4 Hz, 2H), 4.07 (s, 3H); ^13^C NMR (100 MHz, DMSO*-d_6_*) *δ* 175.3, 160.8, 159.4, 149.2, 147.9, 139.8, 133.2, 129.0, 126.7, 126.0, 125.4, 117.7, 115.7, 110.0, 41.3; HRMS (ESI) calcd. for C_18_H_16_N_3_O_3_ [M+H]^+^ 322.1186, found 322.1182.

*Data for* (E)-N'-(4-methoxybenzylidene)-1-methyl-4-oxo-1,4-dihydroquinoline-3-carbohydrazide **(5)***.* White solid; yield: 74.6%. mp 234 - 235 °C. ^1^H NMR (400 MHz, CDCl_3_) *δ* 13.14 (s, 1H), 8.89 (s, 1H), 8.55 (d, *J* = 8.4 Hz, 1H), 8.17 (s, 1H), 7.81 - 7.75 (m, 3H), 7.57 - 7.53 (m, 2H), 6.92 (d, *J* = 8.8 Hz, 2H), 4.01 (s, 3H), 3.84 (s, 3H); ^13^C NMR (100 MHz, CDCl_3_) *δ* 176.6, 161.7, 161.5, 148.9, 148.3, 140.0, 133.3, 129.5, 127.7, 127.4, 126.9, 125.7, 116.1, 114.2, 111.4, 55.5, 41.8; HRMS (ESI) calcd. for C_19_H_18_N_3_O_3_ [M+H]^+^ 336.1343, found 336.1340.

*Data for* (E)-N'-(3,4-dimethoxybenzylidene)-1-methyl-4-oxo-1,4-dihydroquinoline-3-carbohydrazide **(6)**. White solid; yield: 67.1%; mp 283 - 284 °C. ^1^H NMR (400 MHz, DMSO*-d_6_*) *δ* 13.22 (s, 1H), 8.97 (s, 1H), 8.39 (d, *J* = 8.0 Hz, 1H), 8.34 (s, 1H), 7.93 - 7.87 (m, 2H), 7.63 - 7.60 (m, 1H), 7.38 (d, *J* = 2.0 Hz, 1H), 7.27 (dd, *J* = 2.0, 8.4 Hz, 1H), 7.04 (d, *J* = 8.0 Hz, 1H), 4.08 (s, 3H), 3.83 (s, 3H), 3.81 (s, 3H); ^13^C NMR (100 MHz, DMSO*-d_6_*) *δ* 175.4, 160.8, 150.7, 149.2, 149.0, 147.9, 139.9, 133.3, 127.1, 126.7, 126.0, 125.5, 121.9, 117.7, 111.5, 109.9, 108.5, 55.6, 55.4, 41.4; HRMS (ESI) calcd. for C_20_H_20_N_3_O_4_ [M+H]^+^ 366.1448, found 366.1446.

*Data for* (E)-N'-([1,1'-biphenyl]-4-ylmethylene)-1-methyl-4-oxo-1,4-dihydroquinoline-3-carbohydrazide **(7)**. White solid; yield: 89.2%; mp 283 - 284 °C. ^1^H NMR (400 MHz, DMSO*-d_6_*) *δ* 13.34 (s, 1H), 9.01 (s, 1H), 8.48 (s, 1H), 8.41 (d, *J* = 8.0 Hz, 1H), 7.94 - 7.90 (m, 2H), 7.86 (d, *J* = 8.4 Hz, 2H), 7.79 (d, *J* = 8.4 Hz, 2H), 7.74 (d, *J* = 7.6 Hz, 2H), 7.65 - 7.61 (m, 1H), 7.51 -7.48 (m, 2H), 7.42 - 7.38 (m, 1H), 4.09 (s, 3H); ^13^C NMR (100 MHz, DMSO*-d_6_*) *δ* 175.4, 161.1, 149.4, 147.3, 141.5, 139.9, 139.4, 133.5, 133.3, 129.0, 127.9, 127.8, 127.0, 126.7, 126.0, 125.6, 117.8, 109.8, 41.4; HRMS (ESI) calcd. for C_24_H_20_N_3_O_2_ [M+H]^+^ 382.1550, found 382.1548.

*Data for* (E)-1-methyl-4-oxo-N'-(4-phenoxybenzylidene)-1,4-dihydroquinoline-3-carbohydrazide **(8)***.* White solid; yield: 78.1%; mp 249 - 250 °C. ^1^H NMR (400 MHz, DMSO*-d_6_*) *δ* 13.27 (s, 1H), 8.99 (s, 1H), 8.42 (s, 1H), 8.39 (d, *J* = 8.4 Hz, 1H), 7.94 - 7.87 (m, 2H), 7.78 (d, *J* = 8.8 Hz, 2H), 7.64 - 7.60 (m, 1H), 7.46 -7.42 (m, 2H), 7.23 - 7.19 (m, 1H), 7.11 - 7.06 (m, 4H), 4.08 (s, 3H); ^13^C NMR (100 MHz, DMSO*-d_6_*) *δ* 175.4, 161.0, 158.5, 155.8, 149.4, 147.1, 139.9, 133.3, 130.2, 129.4, 129.1, 126.7, 126.0, 125.6, 124.2, 119.4, 118.3, 117.8, 109.9, 41.4; HRMS (ESI) calcd. for C_24_H_20_N_3_O_3_ [M+H]^+^ 398.1499, found 398.1496.

*Data for* (*E*)-1-methyl-N'-(4-(methylthio)benzylidene)-4-oxo-1,4-dihydroquinoline-3-carbohydrazide **(9)**. White solid; yield: 82.9%; mp 263 - 264 °C. ^1^H NMR (400 MHz, CDCl_3_) *δ* 13.21 (s, 1H), 8.89 (s, 1H), 8.55 (d, *J* = 8.4 Hz, 1H), 8.17 (s, 1H), 7.82 - 7.78 (m, 1H), 7.72 (d, *J* = 8.4 Hz, 2H), 7.58 - 7.54 (m, 2H), 7.26 (s, 1H), 7.24 (d, *J* = 8.4 Hz, 1H), 4.01 (s, 3H), 2.51 (s, 3H); ^13^C NMR (100 MHz, CDCl_3_) *δ* 176.7, 161.9, 148.9, 148.0, 141.7, 140.0, 133.4, 130.8, 128.3, 127.7, 127.5, 125.9, 125.8, 116.2, 112.3, 41.8, 15.4; HRMS (ESI) calcd. for C_19_H_18_N_3_O_2_S [M+H]^+^ 352.1114, found 352.1110.

*Data for* (E)-1-methyl-N'-(4-(methylsulfonyl)benzylidene)-4-oxo-1,4-dihydroquinoline-3-carbohydrazide **(10)**. White solid; yield: 86.2%; mp > 300 °C. ^1^H NMR (400 MHz, DMSO*-d_6_*) *δ* 13.47 (s, 1H), 9.02 (s, 1H), 8.56 (s, 1H), 8.40 (d, *J* = 8.0 Hz, 1H), 8.01 (s, 4H), 7.95 - 7.88 (m, 2H), 7.65 - 7.61 (m, 1H), 4.09 (s, 3H), 3.26 (s, 3H); ^13^C NMR (100 MHz, DMSO*-d_6_*) *δ* 175.4, 161.4, 149.6, 145.9, 141.3, 139.9, 139.3, 133.4, 128.0, 127.8, 127.5, 126.7, 126.0, 125.7, 125.5, 117.8, 109.6, 43.5, 41.4; HRMS (ESI) calcd. for C_19_H_18_N_3_O_4_S [M+H]^+^ 384.1013, found 384.1006.

*Data for* (E)-N'-(4-fluorobenzylidene)-1-methyl-4-oxo-1,4-dihydroquinoline-3-carbohydrazide **(11)**. White solid; yield: 78.9%; mp > 300 °C; ^1^H NMR (400 MHz, DMSO*-d_6_*) *δ* 13.30 (s, 1H), 8.99 (s, 1H), 8.44 (s, 1H), 8.39 (d, *J* = 7.6 Hz, 1H), 7.93 - 7.86 (m, 2H), 7.84 -7.80 (m, 2H), 7.63 - 7.60 (m, 1H), 7.33 - 7.29 (m, 2H), 4.08 (s, 3H); ^13^C NMR (100 MHz, DMSO*-d_6_*) *δ* 175.4, 161.1, 149.4, 146.6, 139.9, 133.3, 131.0, 129.4, 129.3, 126.7, 126.0, 125.6, 117.8, 116.0, 115.8, 109.8, 41.4; HRMS (ESI) calcd. for C_18_H_15_FN_3_O_2_ [M+H]^+^ 324.1143, found 324.1141.

*Data for* (E)-N'-(4-chlorobenzylidene)-1-methyl-4-oxo-1,4-dihydroquinoline-3-carbohydrazide **(12)**. White solid; yield: 76.7%; mp > 300 °C. ^1^H NMR (400 MHz, DMSO*-d_6_*) *δ* 13.33 (s, 1H), 8.99 (s, 1H), 8.44 (s, 1H), 8.39 (d, *J* = 8.0 Hz, 1H), 7.93 - 7.87 (m, 2H), 7.78 (d, *J* = 8.4 Hz, 2H), 7.63 - 7.60 (m, 1H), 7.53 (d, *J* = 8.4 Hz, 2H), 4.08 (s, 3H); ^13^C NMR (100 MHz, DMSO*-d_6_*) *δ* 175.4, 161.1, 149.5, 146.4, 139.9, 134.4, 133.4, 133.3, 128.9, 128.8, 126.7, 126.0, 125.6, 117.8, 109.8, 41.4; HRMS (ESI) calcd. for C_18_H_15_ClN_3_O_2_ [M+H]^+^ 340.0847, found 340.0844.

*Data for* (E)-N'-(4-bromobenzylidene)-1-methyl-4-oxo-1,4-dihydroquinoline-3-carbohydrazide **(13)**. White solid; yield: 69.2%; mp > 300 °C. ^1^H NMR (400 MHz, DMSO*-d_6_*) *δ* 13.34 (s, 1H), 9.00 (s, 1H), 8.44 (s, 1H), 8.39 (d, *J* = 7.6 Hz, 1H), 7.94 - 7.87 (m, 2H), 7.73 - 7.60 (m, 6H), 4.08 (s, 3H); ^13^C NMR (100 MHz, DMSO*-d_6_*) *δ* 175.4, 161.1, 149.4, 146.5, 139.9, 133.7, 133.3, 131.8, 129.0, 126.7, 126.0, 125.5, 123.2, 117.7, 109.7, 41.4; HRMS (ESI) calcd. for C_18_H_15_BrN_3_O_2_ [M+H]^+^ 384.0342, found 384.0340.

*Data for* (E)-1-methyl-4-oxo-N'-(4-(trifluoromethoxy)benzylidene)-1,4-dihydroquinoline-3- carbohydrazide **(14)**. White solid; yield: 74.2%; mp 289 - 290 °C. ^1^H NMR (400 MHz, CDCl_3_) *δ* 13.31 (s, 1H), 8.90 (s, 1H), 8.56 (d, *J* = 7.2 Hz, 1H), 8.23 (s, 1H), 7.85 (d, *J* = 8.8 Hz, 2H), 7.81 (d, *J* = 7.6 Hz, 1H), 7.59 - 7.55 (m, 2H), 7.25 - 7.23 (m, 2H), 4.03 (s, 3H); ^13^C NMR (100 MHz, CDCl_3_) *δ* 176.7, 162.1, 150.6, 149.1, 146.7, 140.0, 133.5, 132.9, 129.3, 127.5, 125.9, 121.0, 116.2, 111.2 (1C), 41.9; HRMS (ESI) calcd. for C_19_H_15_F_3_N_3_O_3_ [M+H]^+^ 390.1060, found 390.1056.

*Data for* (E)-1-methyl-4-oxo-N'-(pyridin-4-ylmethylene)-1,4-dihydroquinoline-3-carbohydrazide **(15)**. White solid; yield: 57.3%; mp 295 - 296 °C. ^1^H NMR (400 MHz, DMSO*-d_6_*) *δ* 13.48 (s, 1H), 9.01 (s, 1H), 8.66 (d, *J* = 5.2 Hz, 2H), 8.47 (s, 1H), 8.40 (d, *J* = 8.0 Hz, 1H), 7.94 - 7.88 (m, 2H), 7.68 (d, *J* = 5.6 Hz, 2H), 7.64 - 7.61 (m, 1H), 4.09 (s, 3H); ^13^C NMR (100 MHz, DMSO*-d_6_*) *δ* 175.4, 161.4, 150.2, 149.6, 145.5, 141.6, 139.9, 133.4, 126.7, 126.0, 125.7, 121.0, 117.8, 109.5, 41.4; HRMS (ESI) calcd. for C_17_H_15_N_4_O_2_ [M+H]^+^ 307.1190, found 307.1185.

*Data for* (E)-1-methyl-4-oxo-N'-(pyridin-3-ylmethylene)-1,4-dihydroquinoline-3-carbohydrazide **(16)**. White solid; yield: 80.3%; mp 283 - 284 °C. ^1^H NMR (400 MHz, DMSO*-d_6_*) *δ* 13.41 (s, 1H), 9.00 (s, 1H), 8.89 (s, 1H), 8.61 (d, *J* = 4.0 Hz, 1H), 8.51 (s, 1H), 8.40 (d, *J* = 8.0 Hz, 1H), 8.16 (d, *J* = 8.0 Hz, 1H), 7.94 - 7.88 (m, 2H), 7.63 (dd, *J* = 6.8, 6.8 Hz, 1H), 7.50 (dd, *J* = 4.8, 7.6 Hz, 1H), 4.09 (s, 3H); ^13^C NMR (100 MHz, DMSO*-d_6_*) *δ* 175.4, 161.2, 150.6, 149.5, 148.8, 145.1, 139.9, 133.5, 133.4, 130.3, 126.7, 126.0, 125.6, 124.0, 117.8, 109.6, 41.4; HRMS (ESI) calcd. for C_17_H_15_N_4_O_2_ [M+H]^+^ 307.1190, found 307.1185.

*Data for* (E)-N'-((2,3-dihydrobenzo[b][1,4]dioxin-6-yl)methylene)-1-methyl-4-oxo-1,4-dihydroquinoline-3-carbohydrazide **(17)**. White solid; yield: 84.4%; mp 271 - 272 °C. ^1^H NMR (400 MHz, DMSO*-d_6_*) *δ* 13.20 (s, 1H), 8.97 (s, 1H), 8.39 (d, *J* = 8.0 Hz, 1H), 8.30 (s, 1H), 7.93 - 7.87 (m, 2H), 7.63 - 7.60 (m, 1H), 7.27 - 7.25 (m, 2H), 6.94 (d, *J* = 8.8 Hz, 1H), 4.29 (s, 4H), 4.08 (s, 3H); ^13^C NMR (100 MHz, DMSO*-d_6_*) *δ* 175.3, 160.9, 149.3, 147.3, 145.2, 143.5, 139.8, 133.2, 127.8, 126.7, 126.0, 125.5, 120.7, 117.7, 117.4, 115.6, 109.9, 64.3, 64.0, 41.3; HRMS (ESI) calcd. for C_20_H_18_N_3_O_4_ [M+H]^+^ 364.1292, found 364.1287.

*Data for* (E)-N'-(furan-2-ylmethylene)-1-methyl-4-oxo-1,4-dihydroquinoline-3-carbohydrazide **(18)**. Gray solid; yield: 71.2%; mp 210 - 212 °C. ^1^H NMR (400 MHz, DMSO*-d_6_*) *δ* 13.19 (s, 1H), 8.99 (s, 1H), 8.38 (d, *J* = 8.0 Hz, 1H), 8.34 (s, 1H), 7.93 - 7.87 (m, 3H), 7.63 - 7.60 (m, 1H), 6.89 (d, *J* = 3.2 Hz, 1H), 6.65 (s, 1H), 4.07 (s, 3H); ^13^C NMR (100 MHz, DMSO*-d_6_*) *δ* 175.3, 161.1, 149.5, 149.4, 145.2, 139.9, 137.5, 133.3, 126.7, 126.0, 125.5, 117.8, 113.8, 112.2, 109.8, 41.3; HRMS (ESI) calcd. for C_16_H_14_N_3_O_3_ [M+H]^+^ 296.1030, found 296.1026.

*Data for* (E)-N'-((1H-pyrrol-2-yl)methylene)-1-methyl-4-oxo-1,4-dihydroquinoline-3-carbohydrazide **(19)**. Green solid; yield: 52.7%; mp 290 - 291 °C. ^1^H NMR (400 MHz, DMSO*-d_6_*) *δ* 13.05 (s, 1H), 11.56 (s, 1H), 8.98 (s, 1H), 8.39 (d, *J* = 8.4 Hz, 1H), 8.23 (s, 1H), 7.93 - 7.87 (m, 2H), 7.63 - 7.60 (m, 1H), 6.91 (s, 1H), 6.50 (s, 1H), 6.14 (s, 1H), 4.07 (s, 3H); ^13^C NMR (100 MHz, DMSO*-d_6_*) *δ* 175.3, 160.5, 149.1, 140.7, 139.9, 133.2, 127.0, 126.7, 126.0, 125.4, 122.6, 117.7, 113.8, 110.1, 109.3, 41.3; HRMS (ESI) calcd. for C_16_H_15_N_4_O_2_ [M+H]^+^ 295.1190, found 295.1185.

*Data for* (E)-1-methyl-4-oxo-N'-(thiophen-2-ylmethylene)-1,4-dihydroquinoline-3-carbohydrazide **(20)**. White solid; yield: 74.2%; mp 272 - 273 °C. ^1^H NMR (400 MHz, DMSO*-d_6_*) *δ* 13.24 (s, 1H), 8.97 (s, 1H), 8.67 (s, 1H), 8.39 (d, *J* = 8.0 Hz, 1H), 7.93 - 7.87 (m, 2H), 7.69 (d, *J* = 5.2 Hz, 1H), 7.63 - 7.60 (m, 1H), 7.46 (d, *J* = 2.8 Hz, 1H), 7.15 (dd, *J* = 4.0, 4.0 Hz, 1H), 4.07 (s, 3H); ^13^C NMR (100 MHz, DMSO*-d_6_*) *δ* 175.3, 160.9, 149.3, 142.9, 139.9, 139.0, 133.3, 131.1, 128.9, 127.9, 126.7, 126.0, 125.5, 117.8, 109.8, 41.4; HRMS (ESI) calcd. for C_16_H_14_N_3_O_2_S [M+H]^+^ 312.0801, found 312.0797.

*Data for* (E)-N'-((1H-indol-2-yl)methylene)-1-methyl-4-oxo-1,4-dihydroquinoline-3-carbohydrazide **(21)**. Yellow solid; yield: 89.1%; mp 291 - 292 °C. ^1^H NMR (400 MHz, DMSO*-d_6_*) *δ* 13.30 (s, 1H), 11.60 (s, 1H), 9.01(s, 1H), 8.49 (s, 1H), 8.41 (d, *J* = 8.0 Hz, 1H), 7.94 - 7.87 (m, 2H), 7.64 - 7.61 (m, 1H), 7.57 (d, *J* = 8.0 Hz, 1H), 7.44 (d, *J* = 8.0 Hz, 1H), 7.18 - 7.14 (m, 1H), 7.03 - 6.99 (m, 1H), 6.84 (s, 1H), 4.08 (s, 3H); ^13^C NMR (100 MHz, DMSO*-d_6_*) *δ* 175.4, 161.0, 149.4, 140.6, 139.9, 137.9, 133.3, 133.2, 127.6, 126.7, 126.0, 125.5, 123.3, 120.8, 119.5, 117.8, 112.0, 109.9, 107.2, 41.4; HRMS (ESI) calcd. for C_20_H_17_N_4_O_2_ [M+H]^+^ 345.1346, found 345.1340.

*Data for* (E)-N'-((1H-indol-3-yl)methylene)-1-methyl-4-oxo-1,4-dihydroquinoline-3-carbohydrazide **(22)**. Pink solid; yield: 78.5%; mp > 300 °C. ^1^H NMR (400 MHz, DMSO*-d_6_*) *δ* 13.05 (s, 1H), 11.58 (s, 1H), 8.96 (s, 1H), 8.55 (s, 1H), 8.41 (d, *J* = 8.0 Hz, 1H), 8.28 (d, *J* = 8.0 Hz, 1H), 7.93 - 7.87 (m, 2H), 7.81 (s, 1H), 7.63 - 7.59 (m, 1H), 7.45 (d, *J* = 7.6 Hz, 1H), 7.23 - 7.15 (m, 2H), 4.09 (s, 3H); ^13^C NMR (100 MHz, DMSO*-d_6_*) *δ* 175.3, 160.3, 149.0, 144.6, 139.8, 137.0, 133.1, 130.4, 126.7, 126.0, 125.3, 124.4, 122.6, 121.8, 120.4, 117.6, 111.9, 111.7, 110.4, 41.4; HRMS (ESI) calcd. for C_20_H_17_N_4_O_2_ [M+H]^+^ 345.1346, found 345.1341.

*Data for* (E)-N'-(benzofuran-2-ylmethylene)-1-methyl-4-oxo-1,4-dihydroquinoline-3-carbohydrazide **(23)**. Light yellow solid; yield: 84.1%; mp 272 - 273 °C. ^1^H NMR (400 MHz, DMSO*-d_6_*) *δ* 13.38 (s, 1H), 9.02 (s, 1H), 8.51(s, 1H), 8.39 (d, *J* = 8.0 Hz, 1H), 7.93 - 7.86 (m, 2H), 7.72 (d, *J* = 7.6 Hz, 1H), 7.66 (d, *J* = 8.0 Hz, 1H), 7.64 - 7.60 (m, 1H), 7.41 (dd, *J* = 8.0, 8.0 Hz, 1H), 7.32 (s, 1H), 7.29 (d, J = 7.2 Hz, 1H), 4.08 (s, 3H); ^13^C NMR (100 MHz, DMSO*-d_6_*) *δ* 175.3, 161.3, 154.8, 151.4, 149.6, 139.9, 137.4, 133.3, 127.8, 126.7, 126.1, 126.0, 125.6, 123.5, 121.9, 117.8, 111.4, 110.3, 109.6, 41.3; HRMS (ESI) calcd. for C_20_H_16_N_3_O_3_ [M+H]^+^ 346.1186, found 346.1180.

*Data for* (E)-N'-((1H-benzo[d]imidazol-2-yl)methylene)-1-methyl-4-oxo-1,4-dihydroquinoline-3-carbohydrazide **(24)**. White solid; yield: 72.5%; mp > 300 °C. ^1^H NMR (400 MHz, DMSO*-d_6_*) *δ* 13.49 (s, 1H), 12.99 (s, 1H), 9.04(s, 1H), 8.53 (s, 1H), 8.42 (d, *J* = 8.0 Hz, 1H), 7.95 - 7.89 (m, 2H), 7.68 - 7.62 (m, 2H), 7.51 (d, *J* = 7.6 Hz, 1H), 7.28 - 7.18 (m, 2H), 4.09 (s, 3H); ^13^C NMR (100 MHz, DMSO*-d_6_*) *δ* 175.4, 161.6, 149.7, 148.1, 143.7, 139.9, 139.2, 134.7, 133.4, 126.7, 126.0, 125.6, 123.8, 121.9, 119.3, 117.8, 112.0, 109.5, 41.4; HRMS (ESI) calcd. for C_19_H_16_N_5_O_2_ [M+H]^+^ 346.1299, found 346.1294.

*Data for* (E)-N'-((9-ethyl-9H-carbazol-2-yl)methylene)-1-methyl-4-oxo-1,4-dihydroquinoline-3-carbohydrazide **(25)**. Light yellow solid; yield: 88.9%; mp 295 - 296 °C. ^1^H NMR (400 MHz, DMSO*-d_6_*) *δ* 13.26 (s, 1H), 9.01 (s, 1H), 8.54 (d, *J* = 14.0 Hz, 2H), 8.42 (d, *J* = 7.6 Hz, 1H), 8.22 (d, *J* = 7.6 Hz, 1H), 7.94 - 7.87 (m, 3H), 7.71 (d, *J* = 8.8 Hz, 1H), 7.67 - 7.61 (m, 2H), 7.52 - 7.48 (m, 1H), 7.28 - 7.24 (m, 1H), 4.48 (q, *J* = 6.8 Hz, 2H), 4.09 (s, 3H), 1.34 (t, *J* = 6.8 Hz, 3H); ^13^C NMR (100 MHz, DMSO*-d_6_*) *δ* 175.4, 160.8, 149.2, 148.8, 140.7, 140.0, 139.9, 133.2, 126.7, 126.2, 126.0, 125.5, 125.3, 125.0, 122.3, 122.1, 120.6, 120.0, 119.4, 117.7, 110.0, 109.5, 41.4, 37.2, 13.7; HRMS (ESI) calcd. for C_26_H_23_N_4_O_2_ [M+H]^+^ 423.1816, found 423.1808.

*Data for* (E)-1-methyl-4-oxo-N'-(quinolin-8-ylmethylene)-1,4-dihydroquinoline-3-carbohydrazide **(26)***.* White solid; yield: 90.1%; mp > 300 °C. ^1^H NMR (400 MHz, DMSO*-d_6_*) *δ* 13.54 (s, 1H), 9.50 (s, 1H), 9.00 (m, 2H), 8.45 (d, *J* = 8.0 Hz, 1H), 8.41 - 8.37 (m, 2H), 8.09 (d, *J* = 8.0 Hz, 1H), 7.92 - 7.86 (m, 2H), 7.72 (dd, *J* = 7.6, 7.6 Hz, 1H), 7.65 - 7.60 (m, 2H), 4.08 (s, 3H); ^13^C NMR (100 MHz, DMSO*-d_6_*) *δ* 175.5, 161.1, 150.5, 149.4, 145.3, 144.0, 139.9, 136.6, 133.3, 130.9, 130.1, 128.0, 126.7, 126.5, 126.0, 125.8, 125.6, 122.0, 117.7, 109.8, 41.4; HRMS (ESI) calcd. for C_21_H_17_N_4_O_2_ [M+H]^+^ 357.1346, found 357.1341.

*Data for* (E)-N'-(anthracen-9-ylmethylene)-1-methyl-4-oxo-1,4-dihydroquinoline-3-carbohydrazide **(27)**. Yellow solid; yield: 81.5%; mp > 300 °C. ^1^H NMR (400 MHz, DMSO*-d_6_*) *δ* 13.51 (s, 1H), 9.57 (s, 1H), 9.03 (s, 1H), 8.77 (d, *J* = 8.4 Hz, 2H), 8.73 (s, 1H), 8.45 (d, *J* = 7.6 Hz, 1H), 8.16 (d, *J* = 7.6 Hz, 2H), 7.91 (m, 2H), 7.64 - 7.57 (m, 5H), 4.10 (s, 3H); ^13^C NMR (100 MHz, DMSO*-d_6_*) *δ* 175.3, 161.3, 149.6, 146.4, 139.9, 133.3, 130.9, 129.7, 129.3, 128.9, 127.0, 126.8, 126.0, 125.8, 125.6, 125.5, 125.3, 117.8, 110.0, 41.4; HRMS (ESI) calcd. for C_26_H_20_N_3_O_2_ [M+H]^+^ 406.1550, found 406.1546.

**^1^H NMR and ^13^C NMR spectrum for echinopsine and compounds 1-27:**


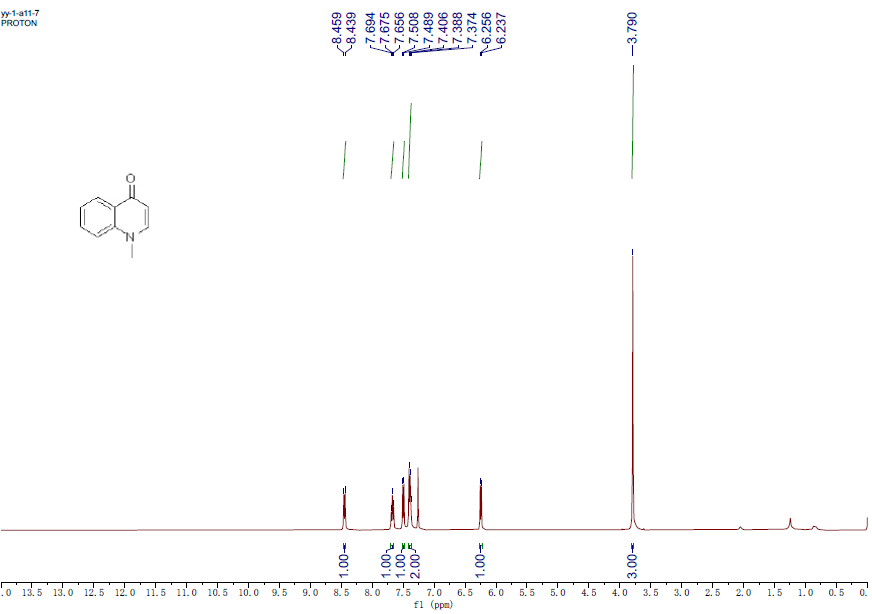


**Fig. S1** ^1^H NMR spectrum of **echinopsine**


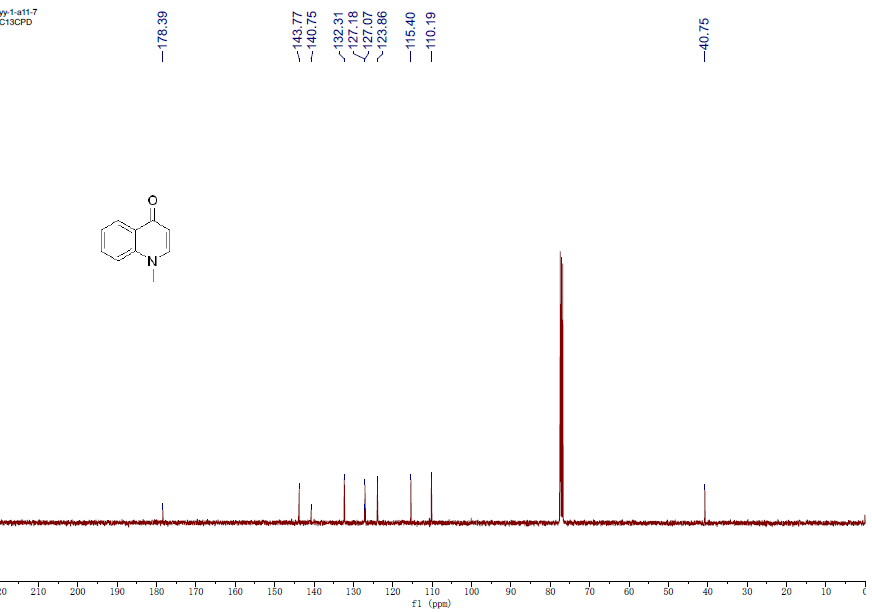


**Fig. S2** ^13^C NMR spectrum of **echinopsine**


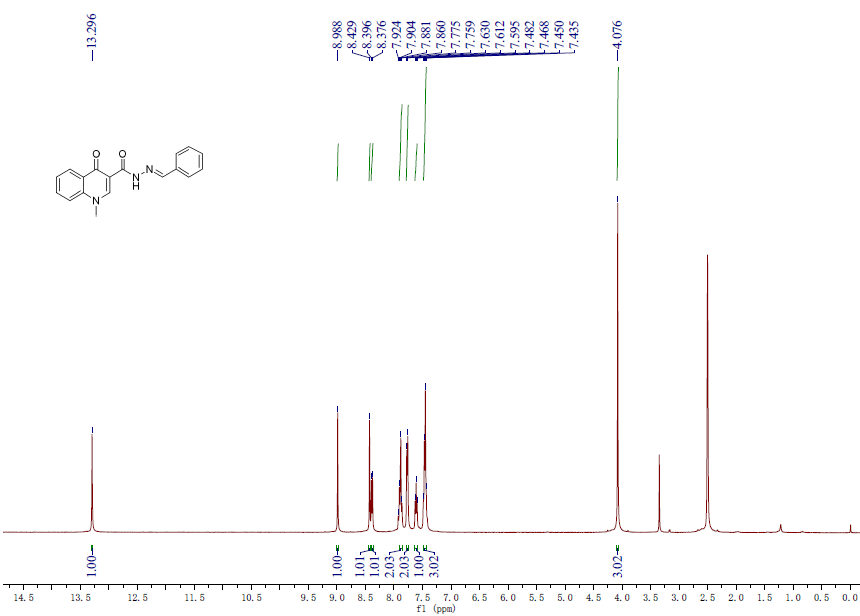


**Fig. S3** ^1^H NMR spectrum of **1**


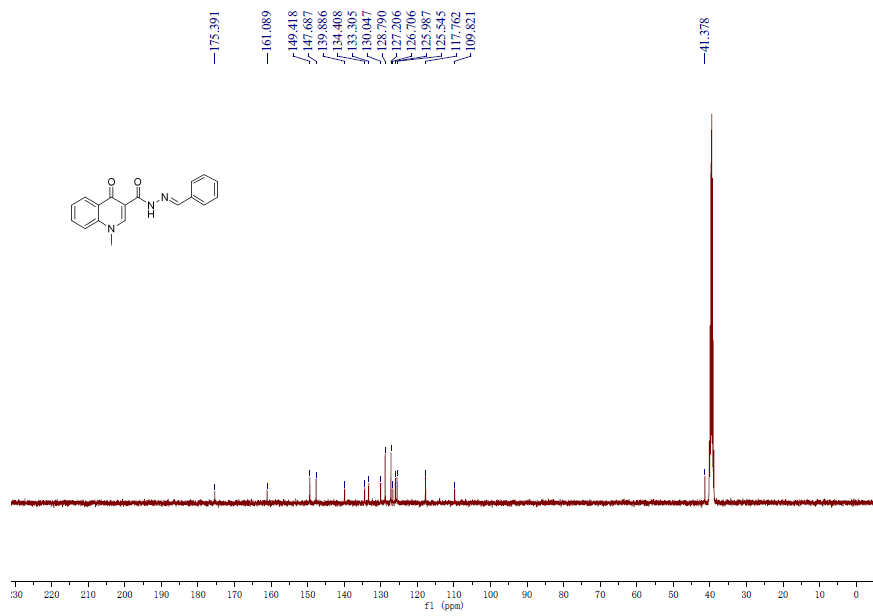


**Fig. S4** ^13^C NMR spectrum of **1**


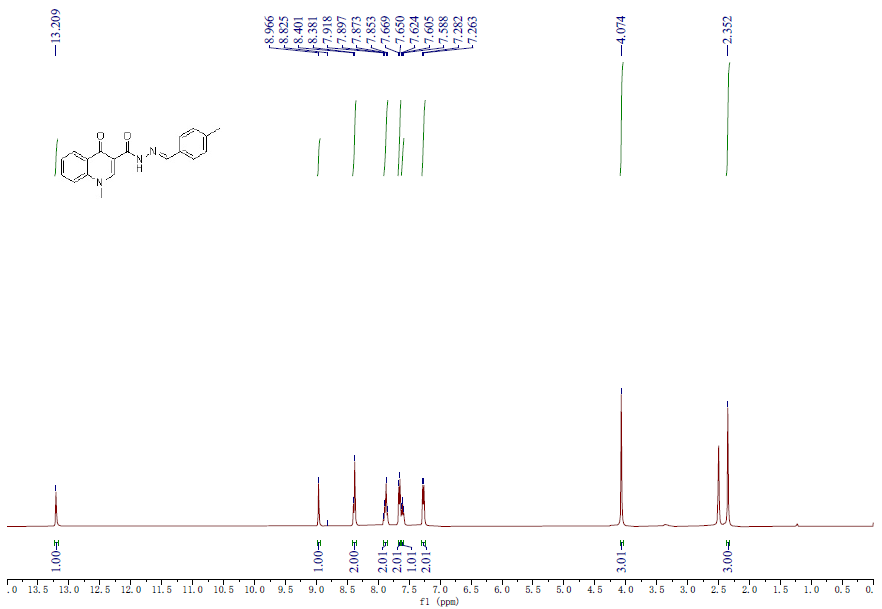


**Fig. S5** ^1^H NMR spectrum of **2**


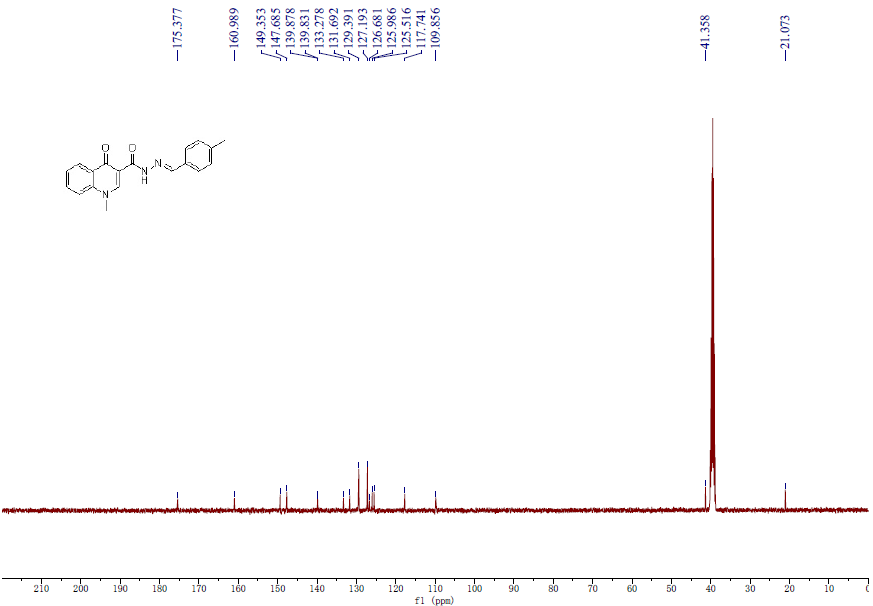


**Fig. S6** ^13^C NMR spectrum of **2**


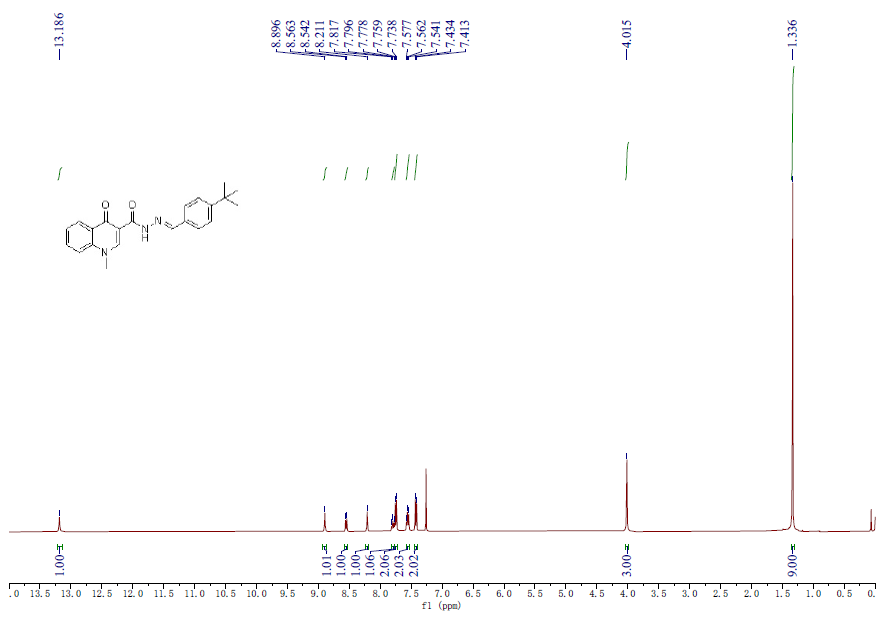


**Fig. S7** ^1^H NMR spectrum of **3**


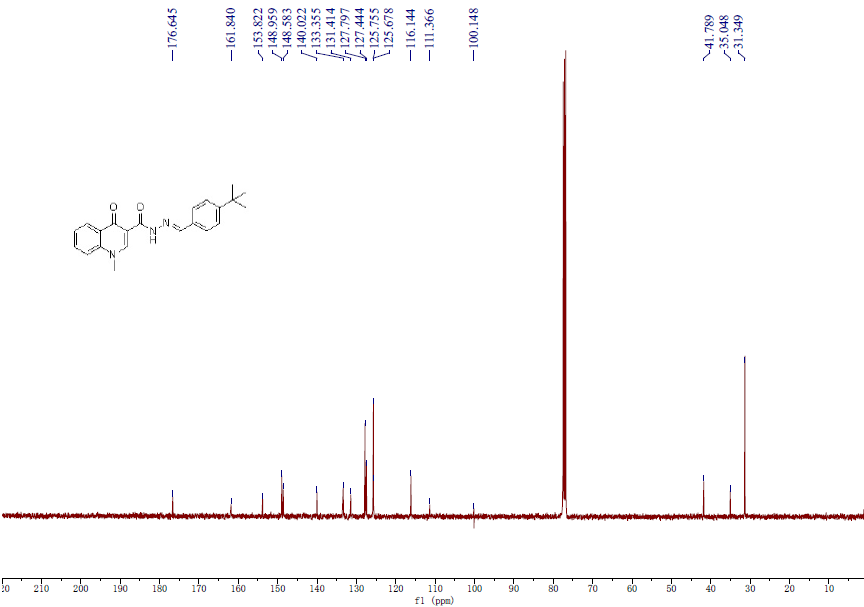


**Fig. S8** ^13^C NMR spectrum of **3**


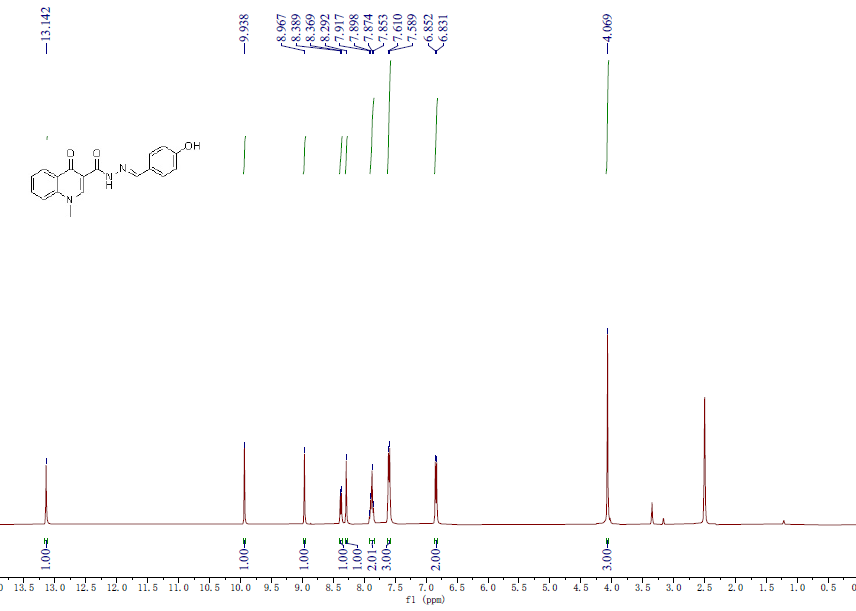


**Fig. S9** ^1^H NMR spectrum of **4**


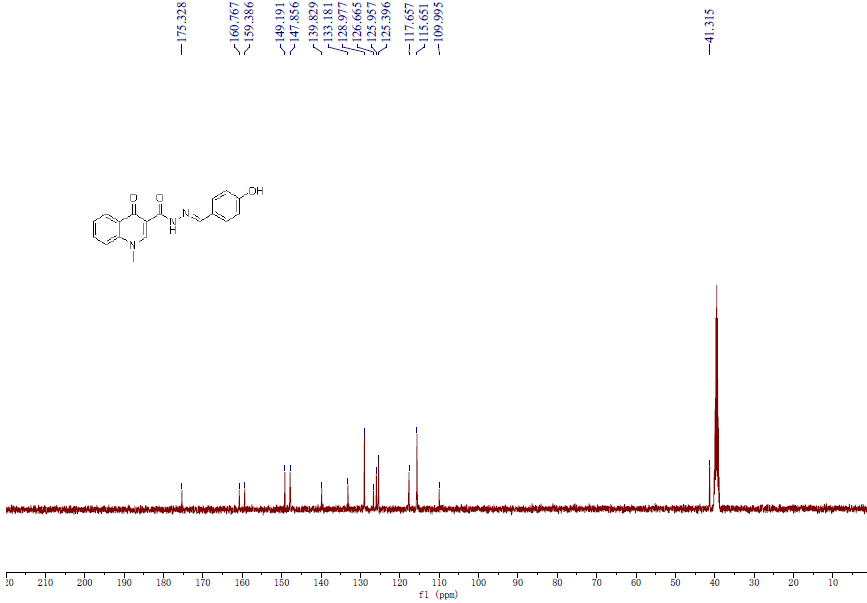


**Fig. S10** ^13^C NMR spectrum of **4**


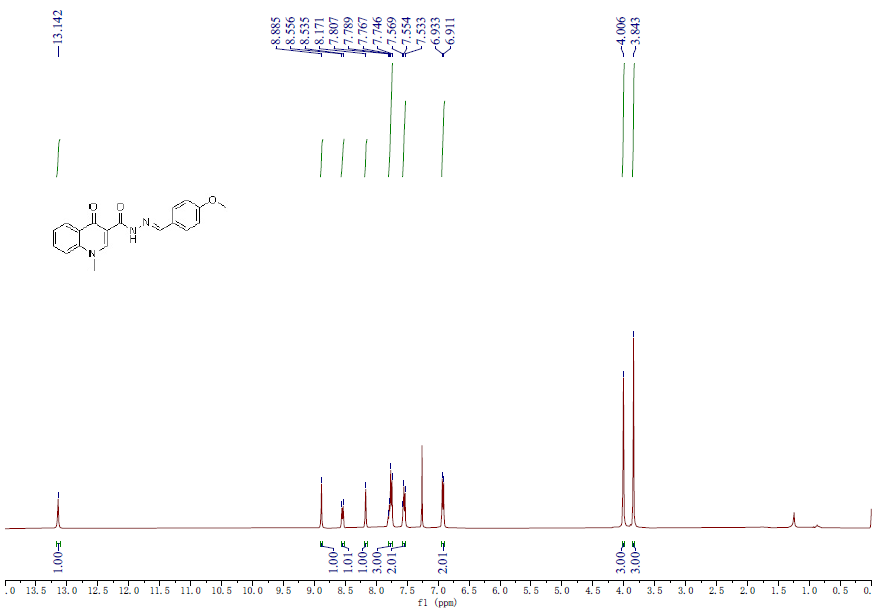


**Fig. S11** ^1^H NMR spectrum of **5**


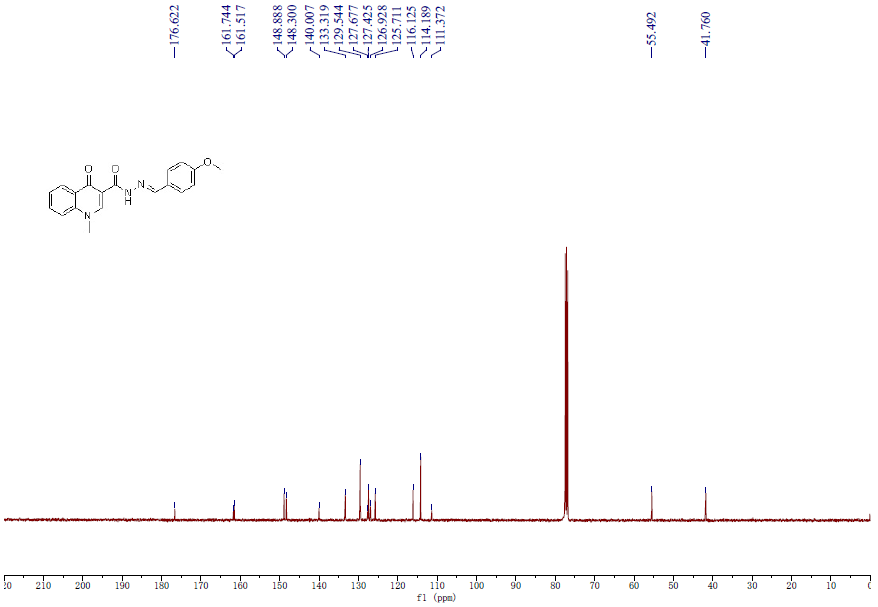


**Fig. S12** ^13^C NMR spectrum of **5**


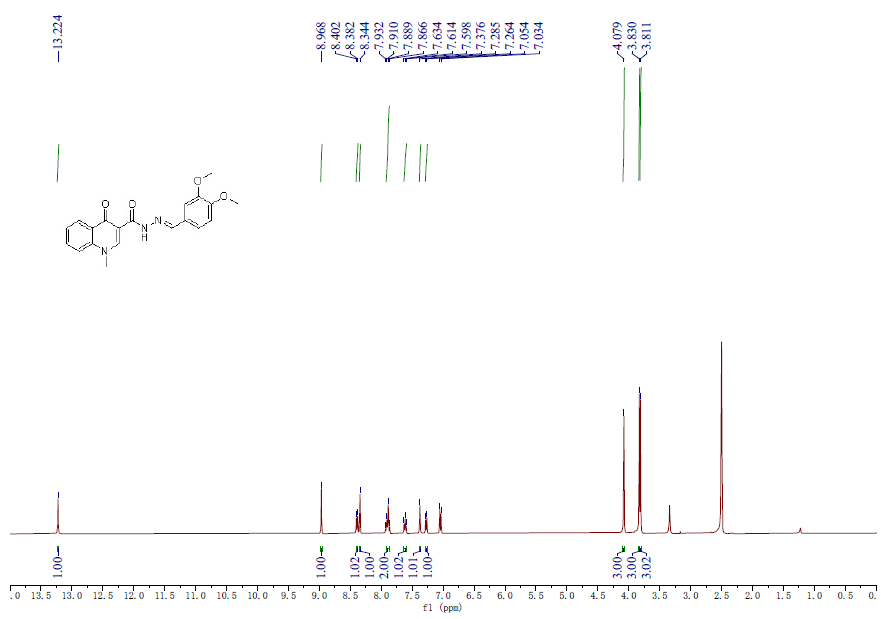


**Fig. S13** ^1^H NMR spectrum of **6**


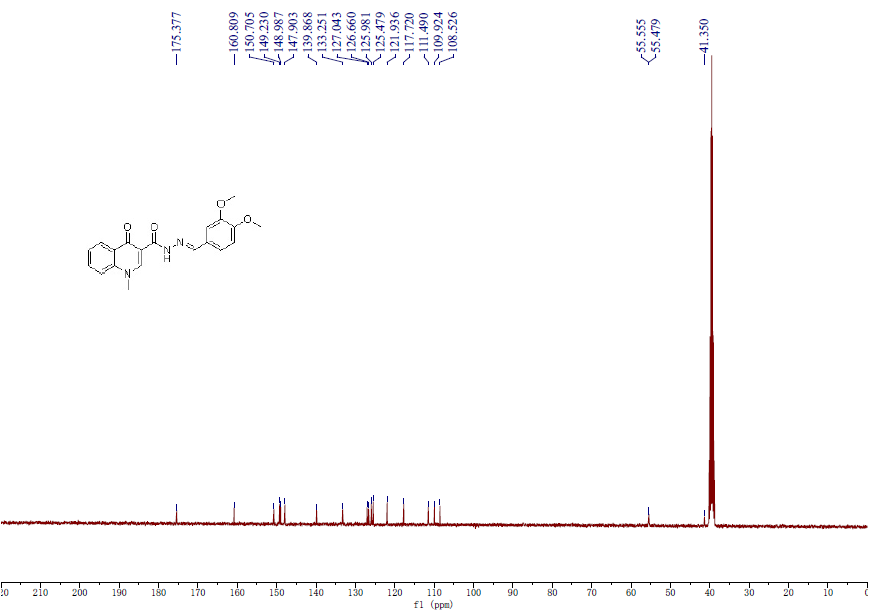


**Fig. S14** ^13^C NMR spectrum of **6**


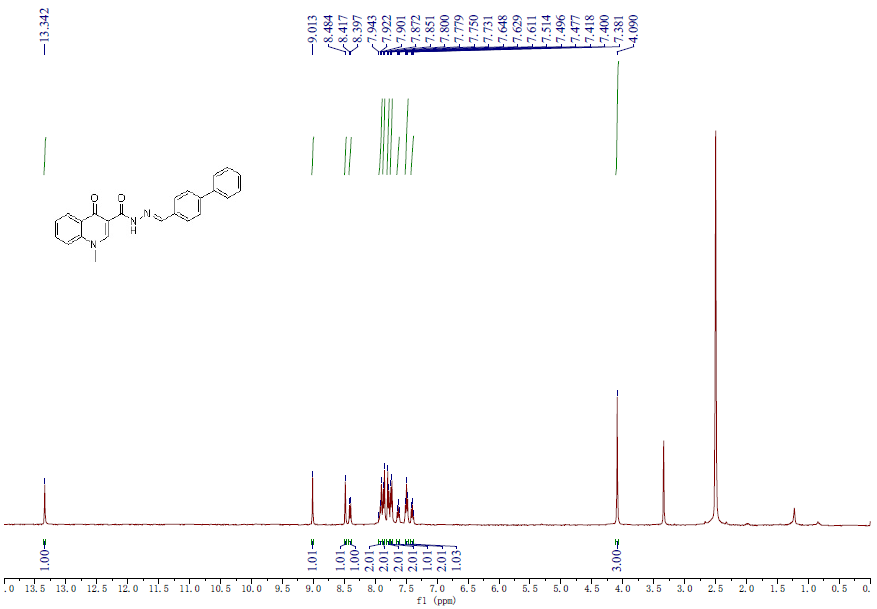


**Fig. S15** ^1^H NMR spectrum of **7**


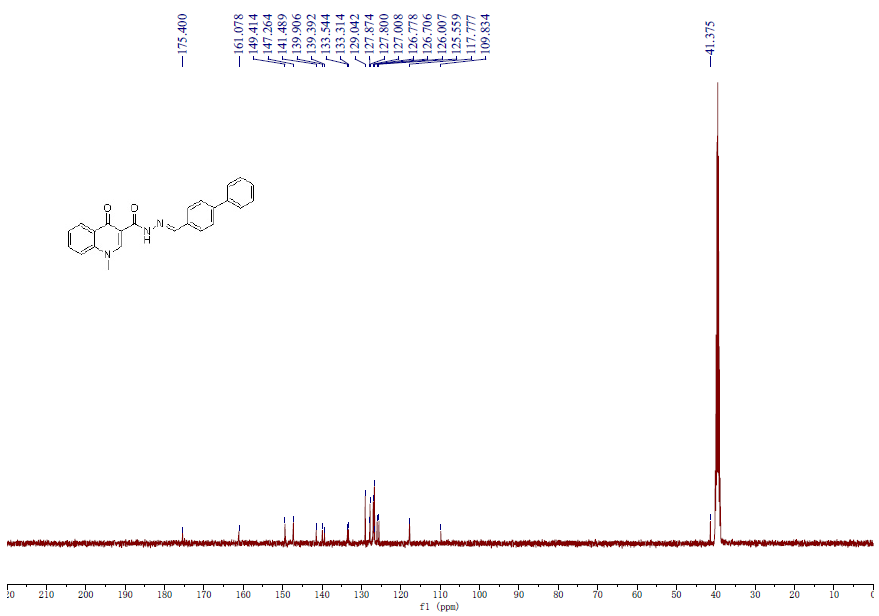


**Fig. S16** ^13^C NMR spectrum of **7**


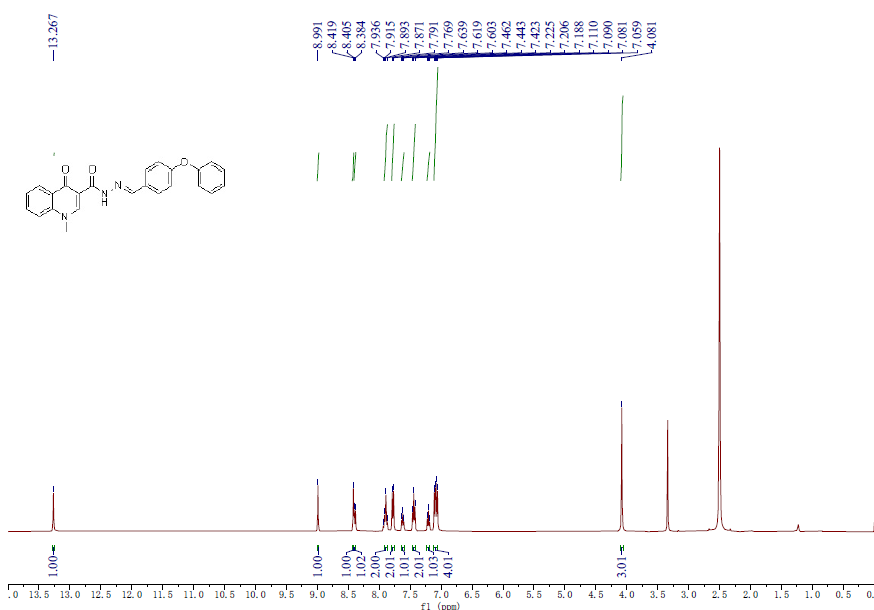


**Fig. S17** ^1^H NMR spectrum of **8**


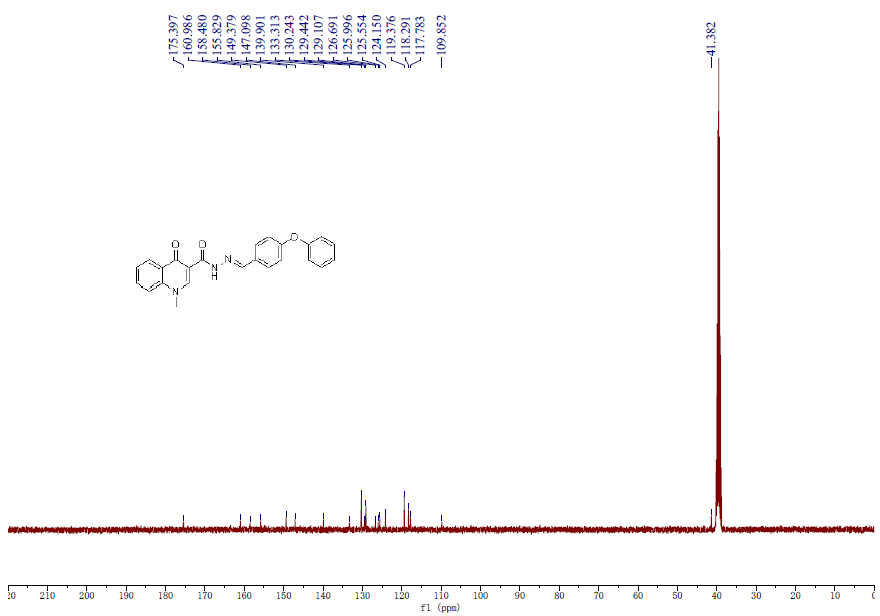


**Fig. S18** ^13^C NMR spectrum of **8**


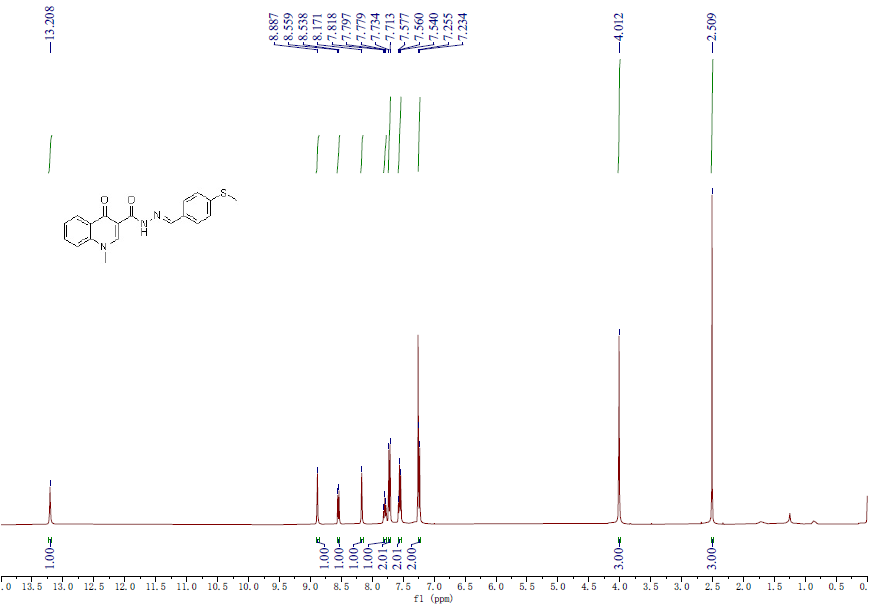


**Fig. S19** ^1^H NMR spectrum of **9**


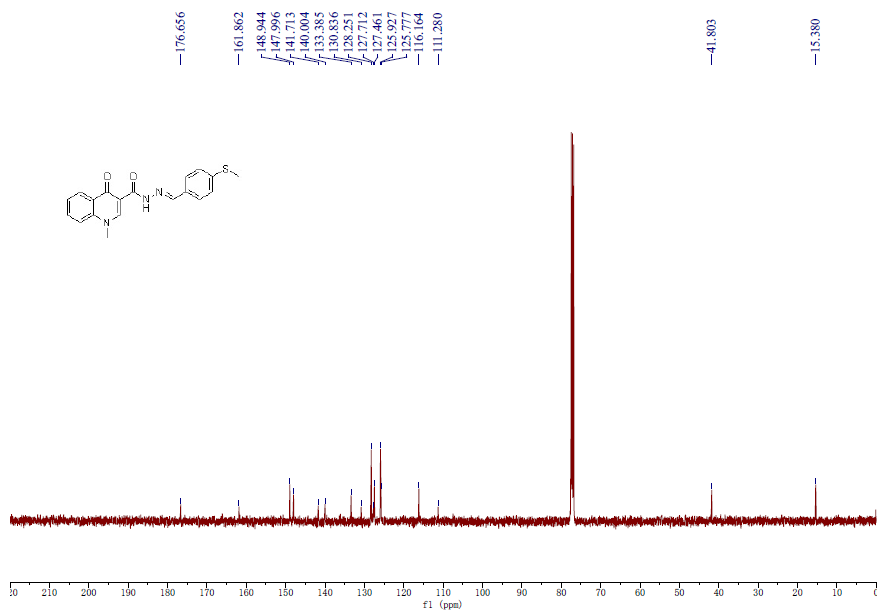


**Fig. S20** ^13^C NMR spectrum of **9**


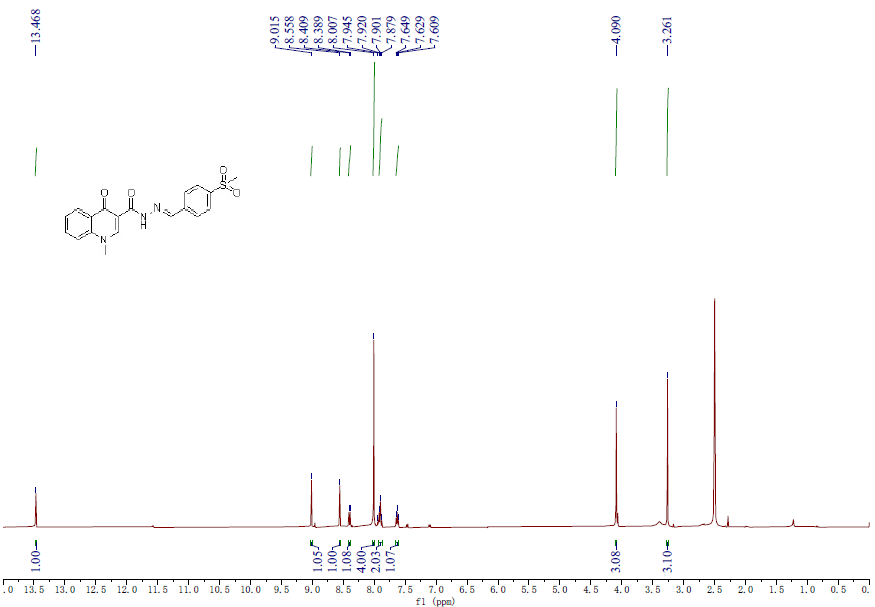


**Fig. S21** ^1^H NMR spectrum of **10**


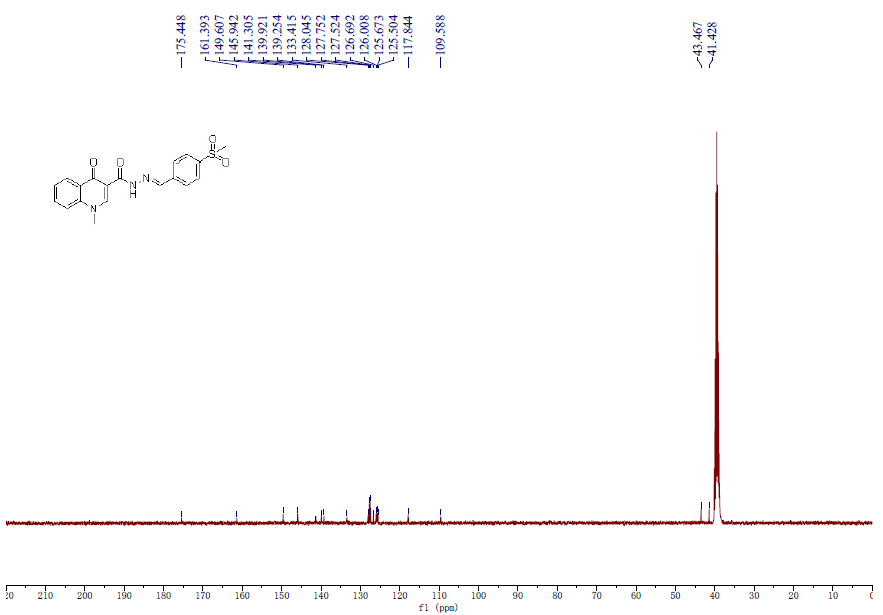


**Fig. S22** ^13^C NMR spectrum of **10**


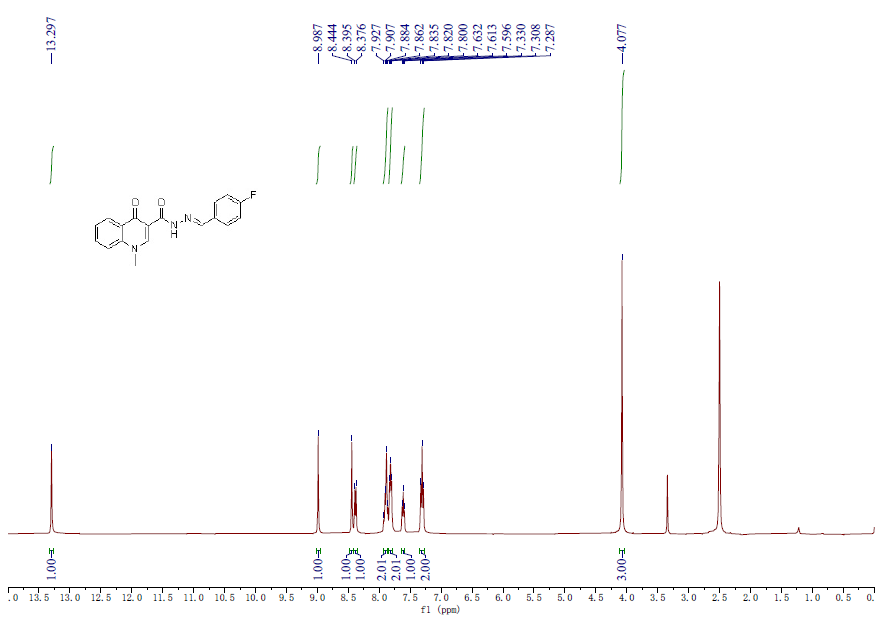


**Fig. S23** ^1^H NMR spectrum of **11**


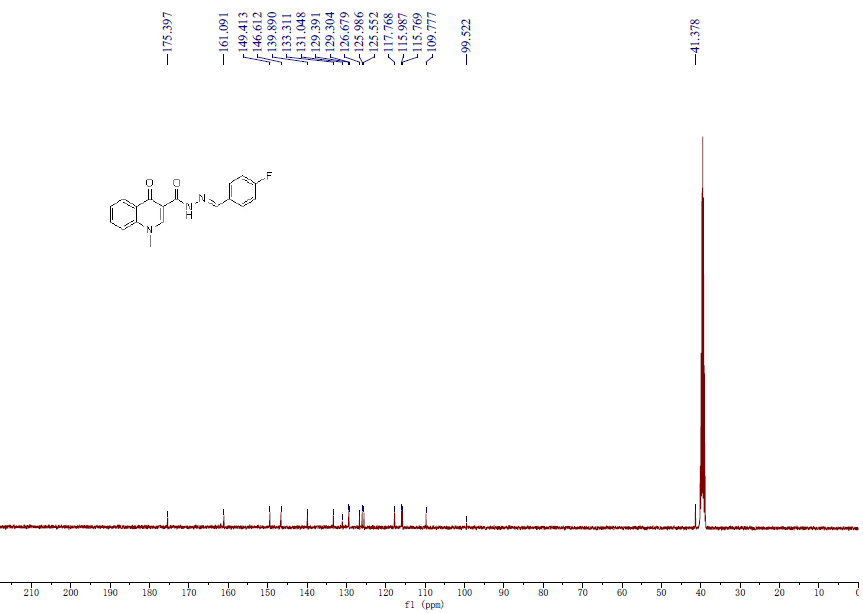


**Fig. S24** ^13^C NMR spectrum of **11**


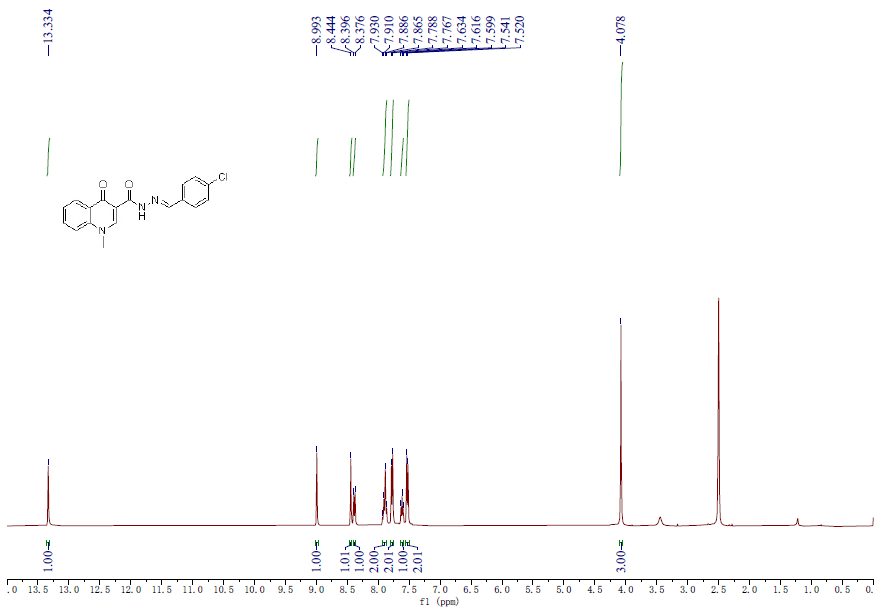


**Fig. S25** ^1^H NMR spectrum of **12**


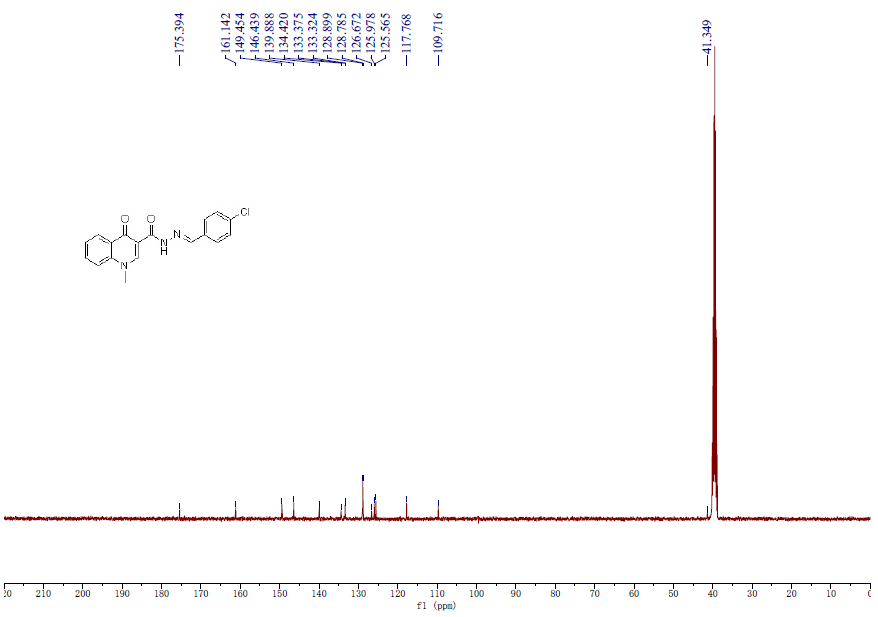


**Fig. S26** ^13^C NMR spectrum of **12**


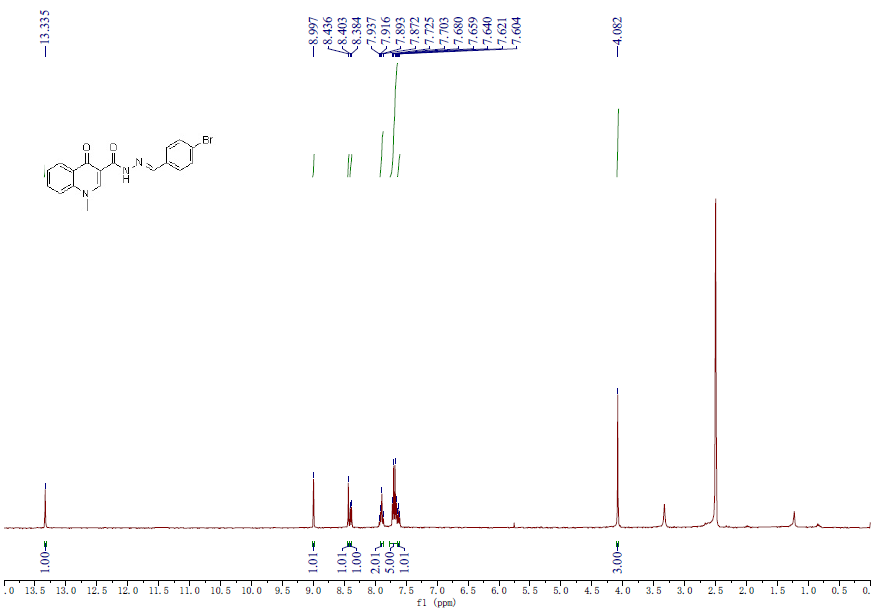


**Fig. S27** ^1^H NMR spectrum of **13**


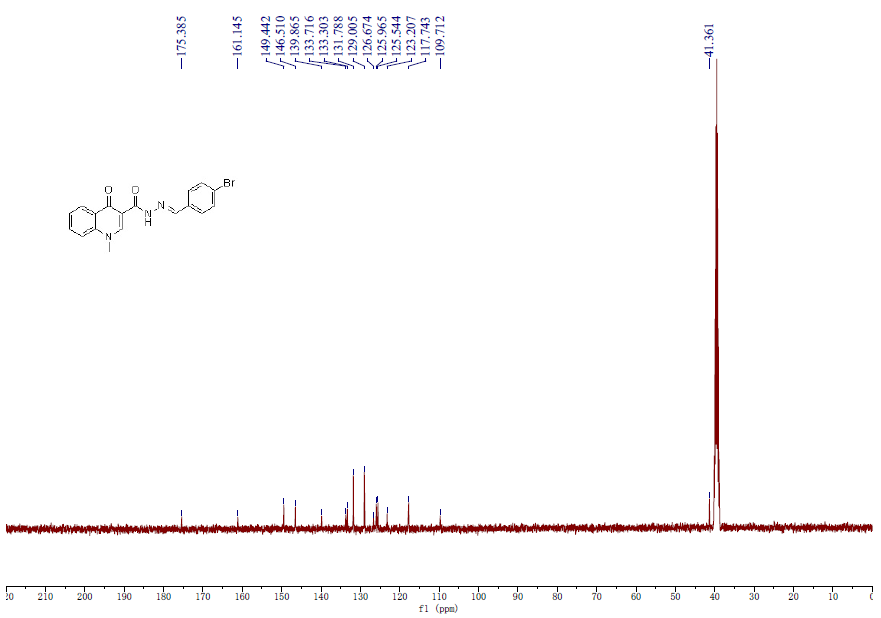


**Fig. S28** ^13^C NMR spectrum of **13**


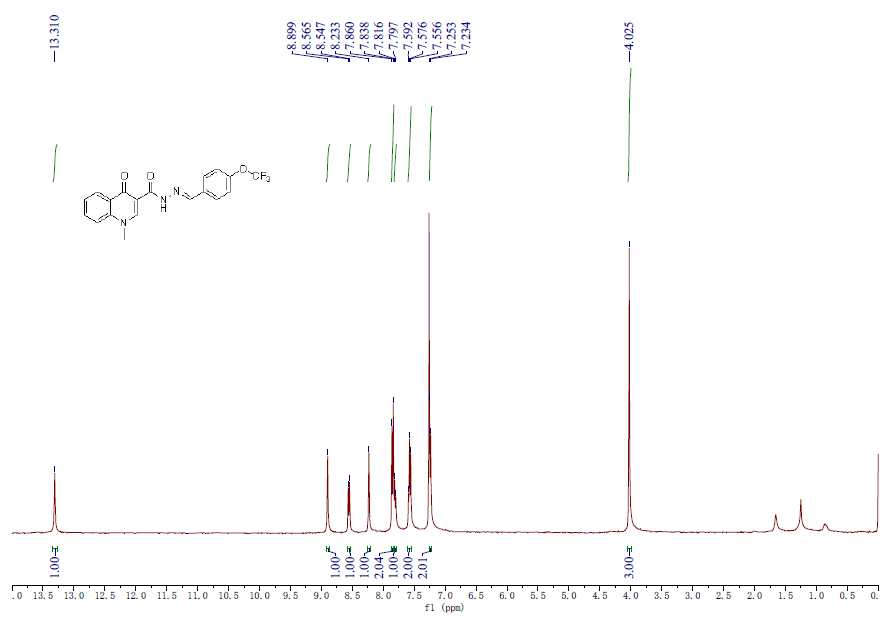


**Fig. S29** ^1^H NMR spectrum of **14**


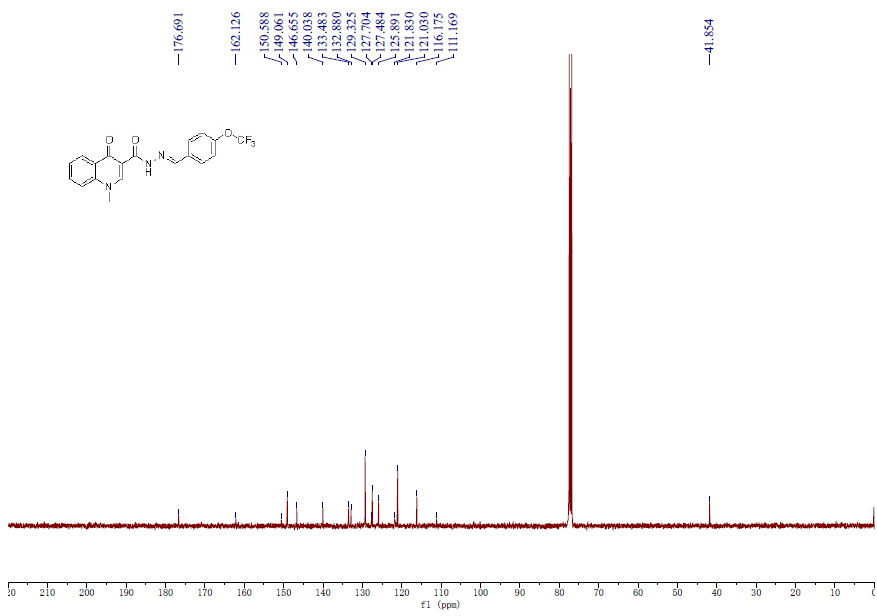


**Fig. S30** ^13^C NMR spectrum of **14**


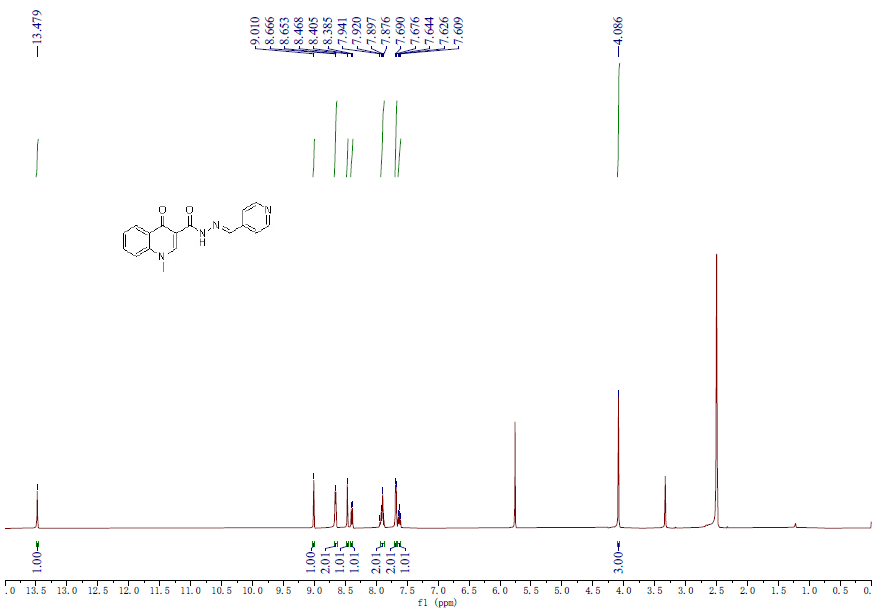


**Fig. S31** ^1^H NMR spectrum of **15**


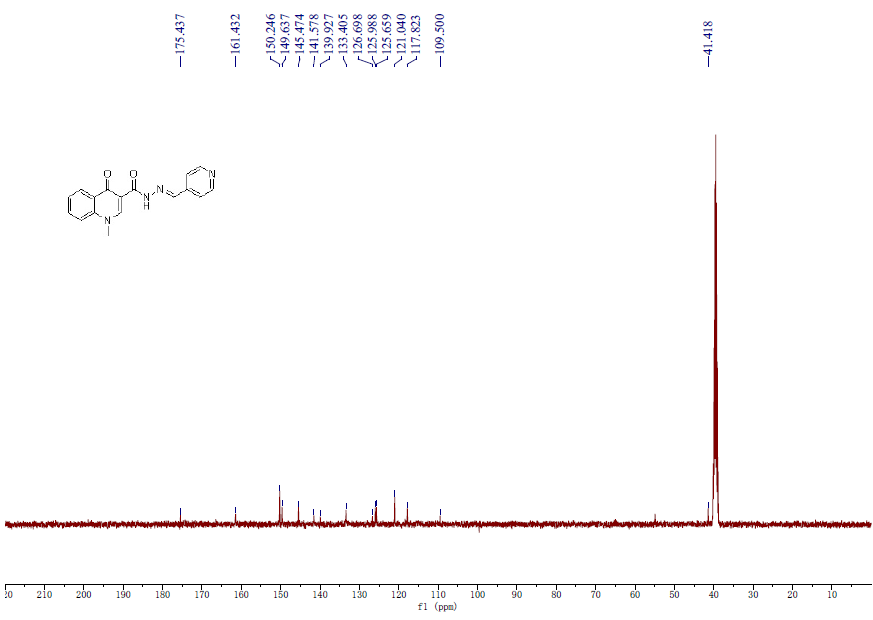


**Fig. S32** ^13^C NMR spectrum of **15**


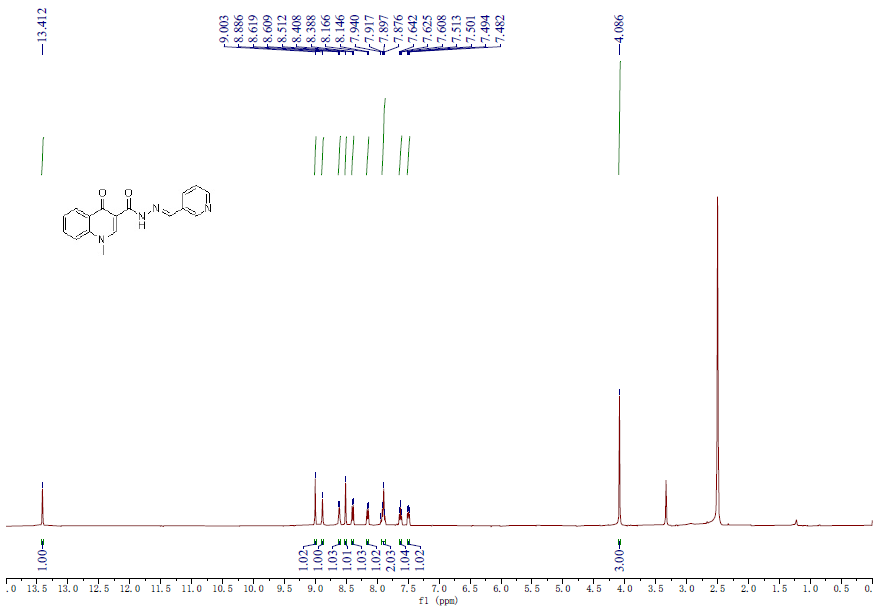


**Fig. S33** ^1^H NMR spectrum of **16**


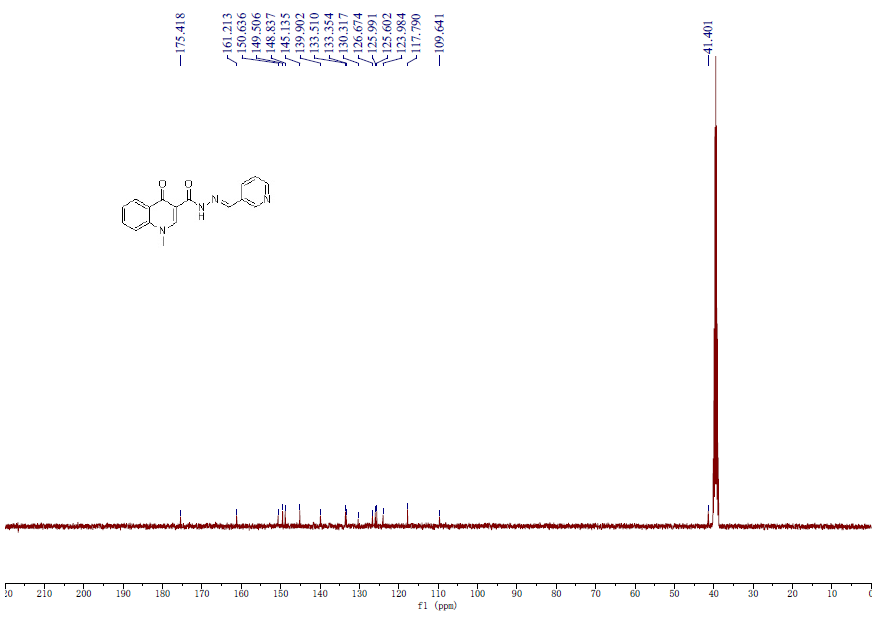


**Fig. S34** ^13^C NMR spectrum of **16**


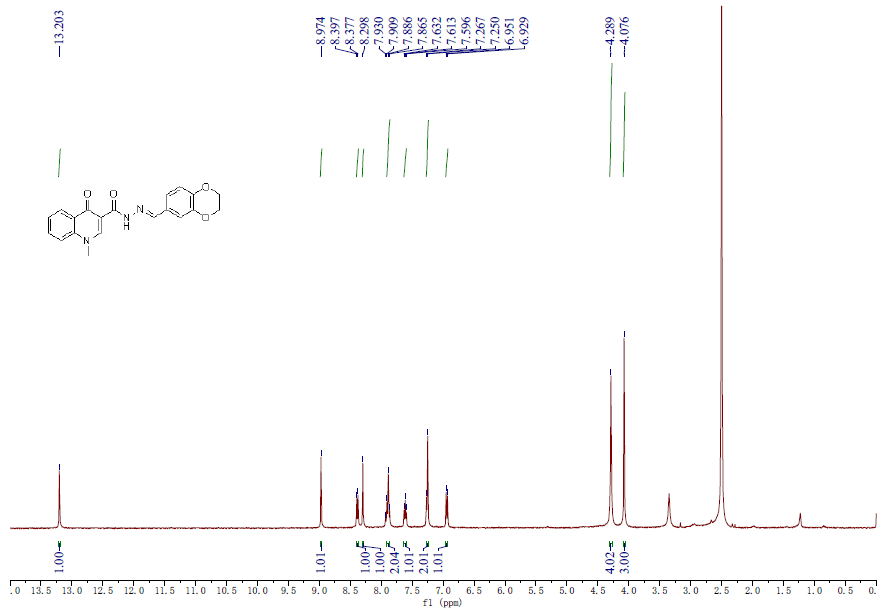


**Fig. S35** ^1^H NMR spectrum of **17**


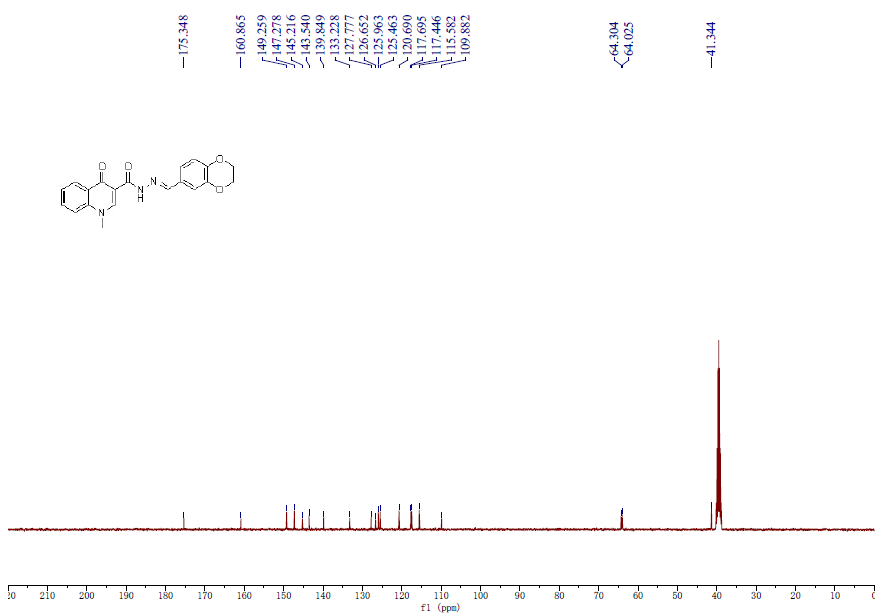


**Fig. S36** ^13^C NMR spectrum of **17**


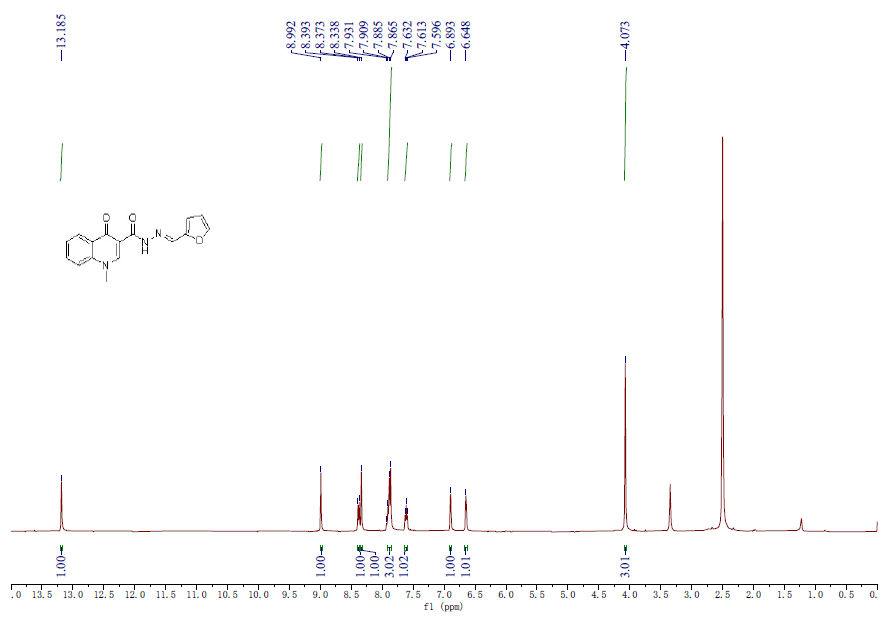


**Fig. S37** ^1^H NMR spectrum of **18**


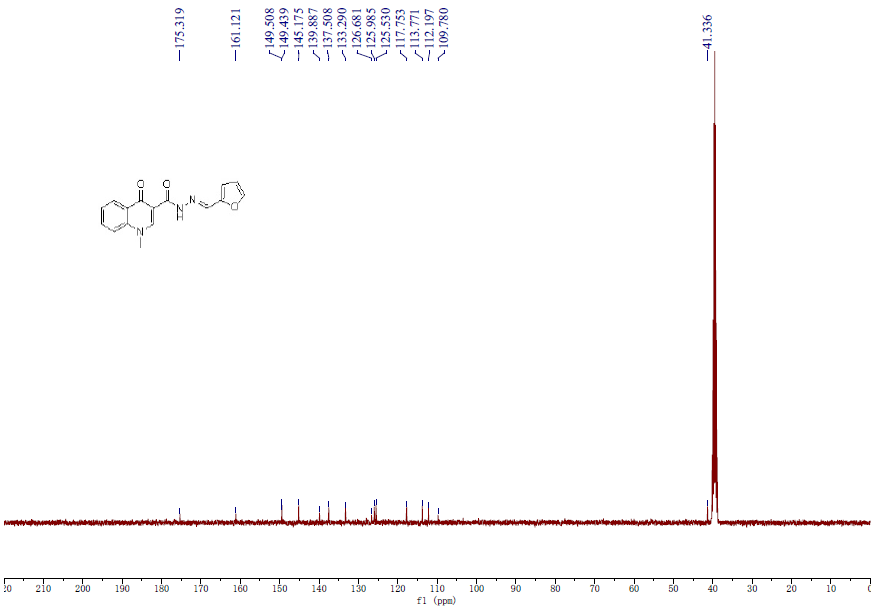


**Fig. S38** ^13^C NMR spectrum of **18**


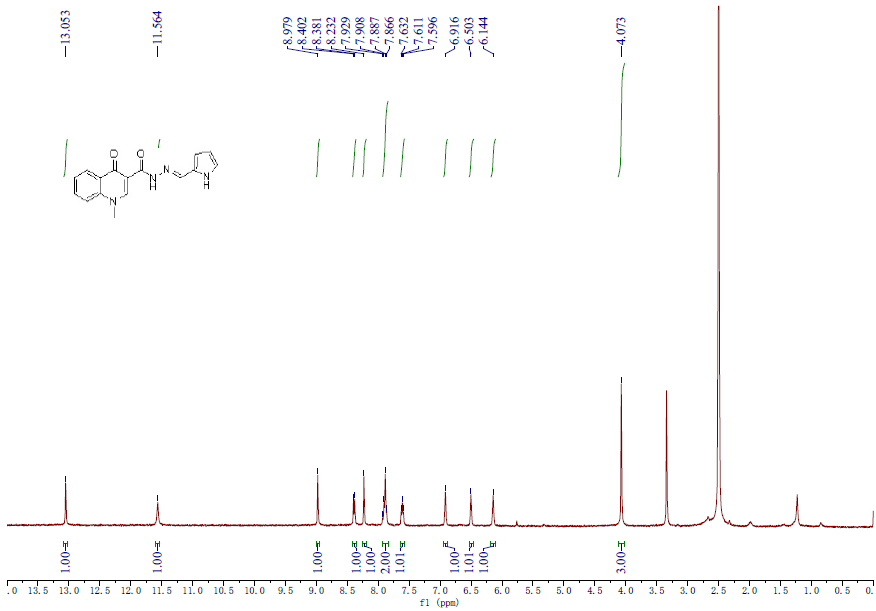


**Fig. S39** ^1^H NMR spectrum of **19**


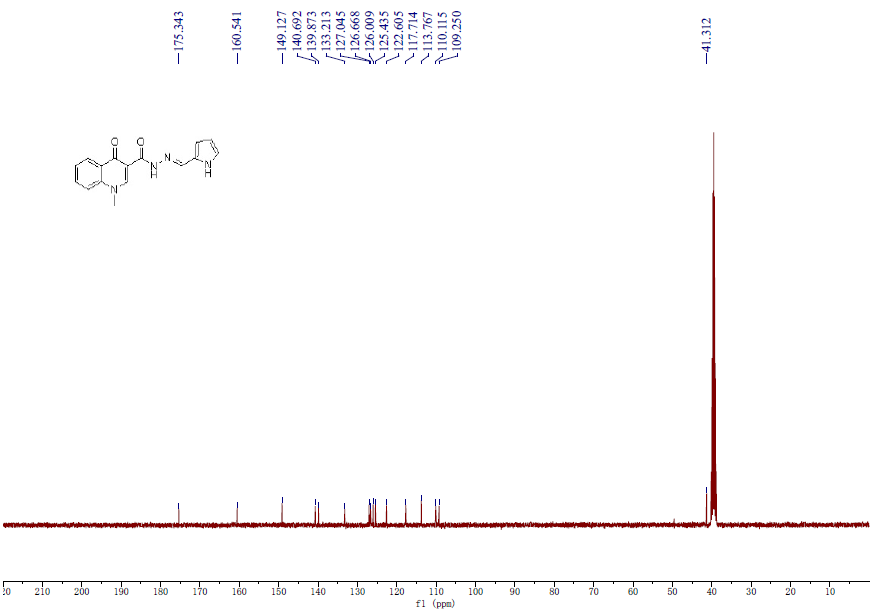


**Fig. S40** ^13^C NMR spectrum of **19**


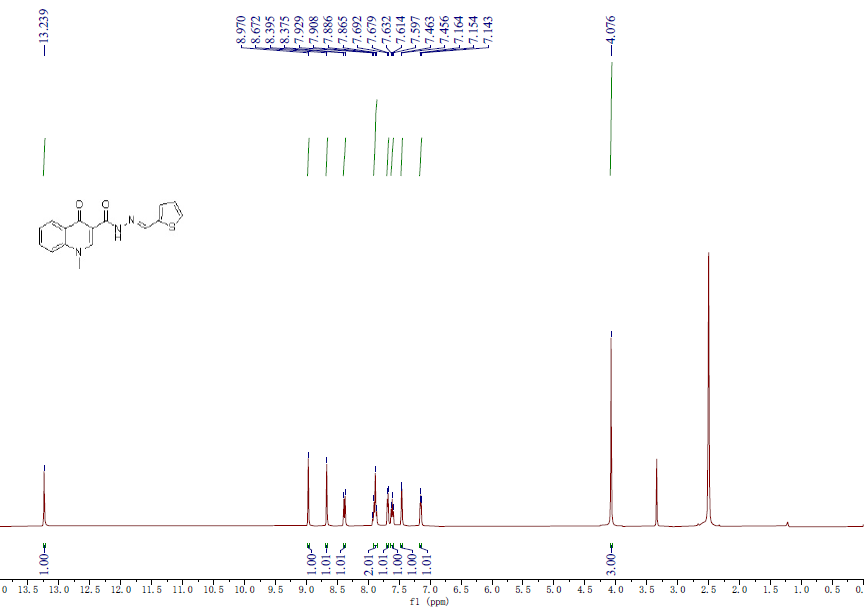


**Fig. S41** ^1^H NMR spectrum of **20**


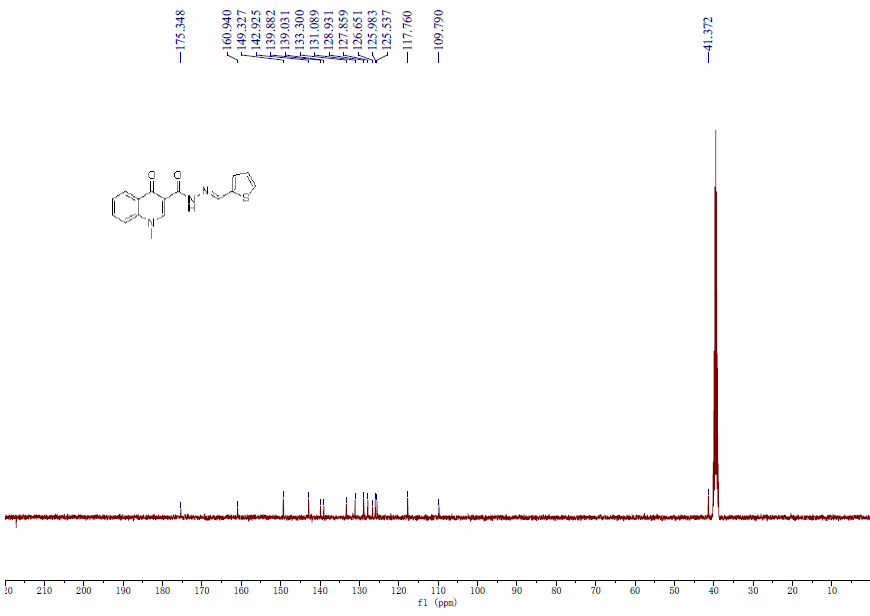


**Fig. S42** ^13^C NMR spectrum of **20**


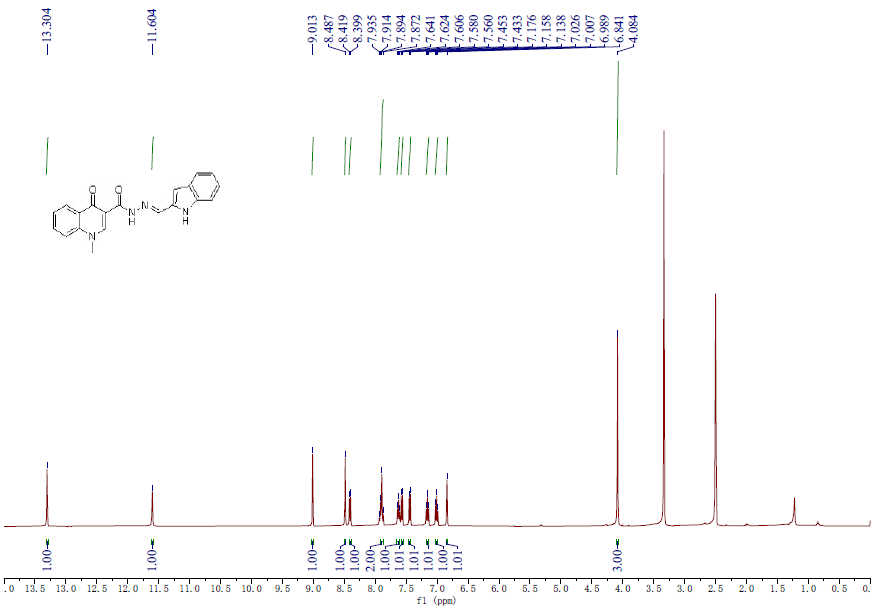


**Fig. S43** ^1^H NMR spectrum of **21**


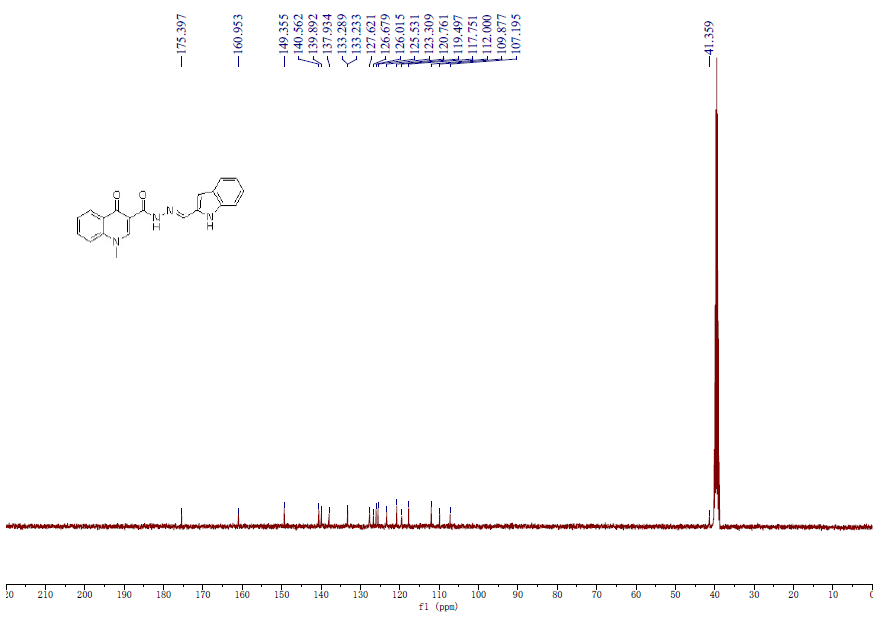


**Fig. S44** ^13^C NMR spectrum of **21**


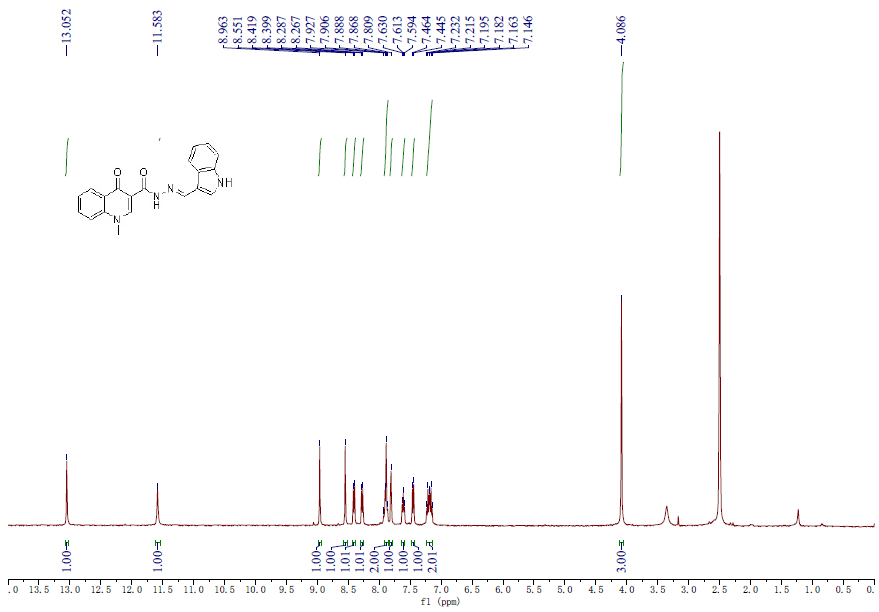


**Fig. S45** ^1^H NMR spectrum of **22**


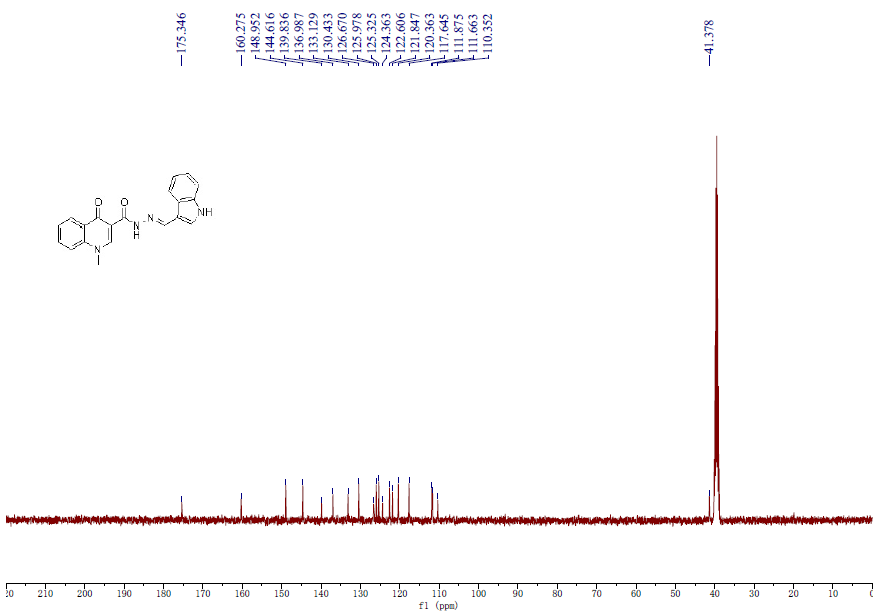


**Fig. S46** ^13^C NMR spectrum of **22**


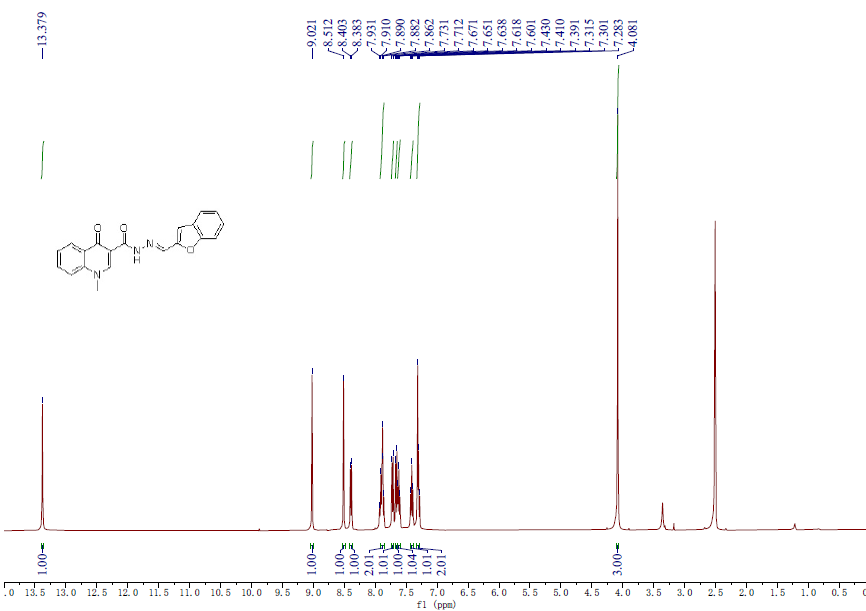


**Fig. S47** ^1^H NMR spectrum of **23**


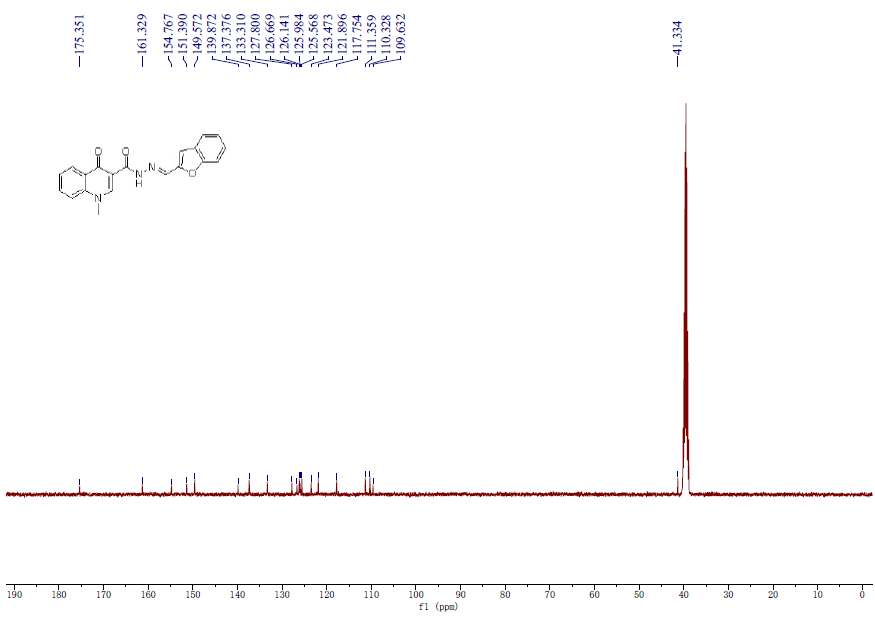


**Fig. S48** ^13^C NMR spectrum of **23**


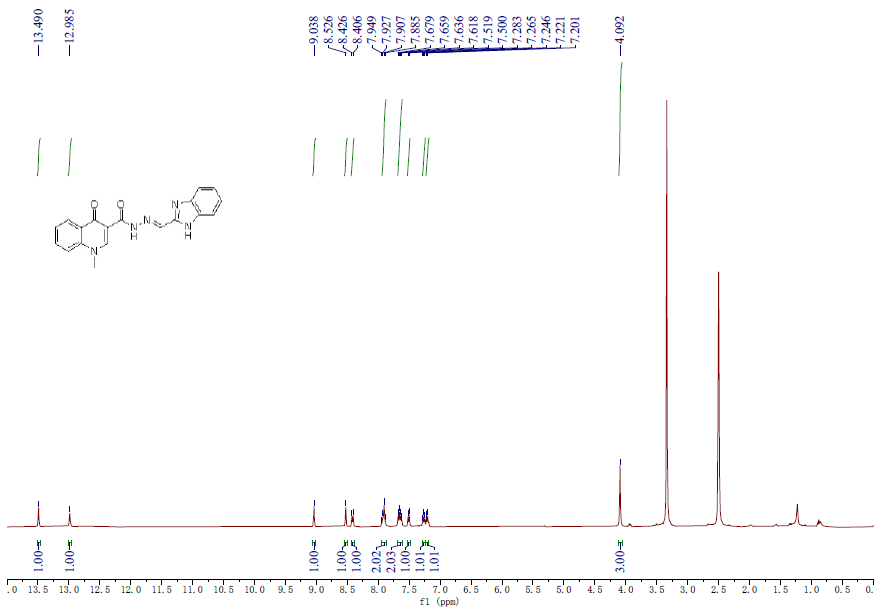


**Fig. S49** ^1^H NMR spectrum of **24**


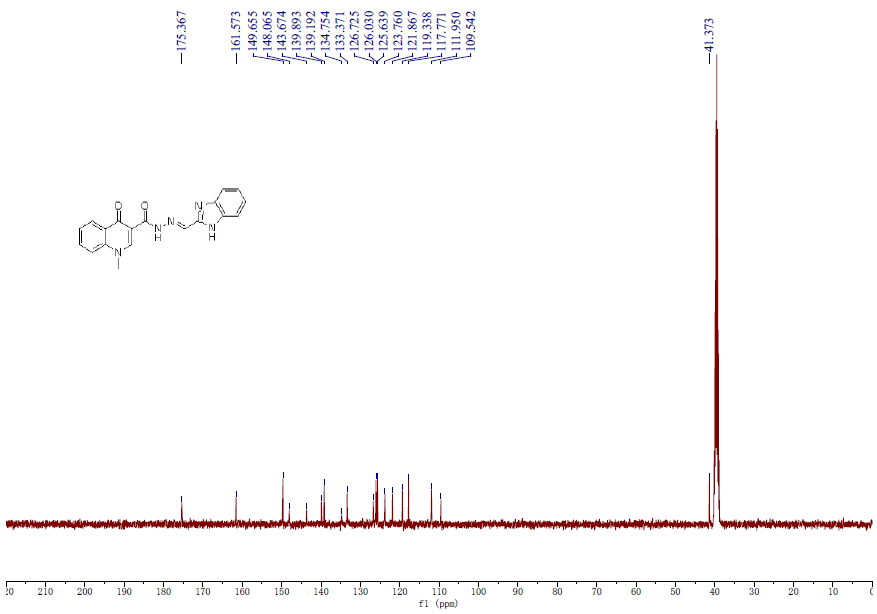


**Fig. S50** ^13^C NMR spectrum of **24**


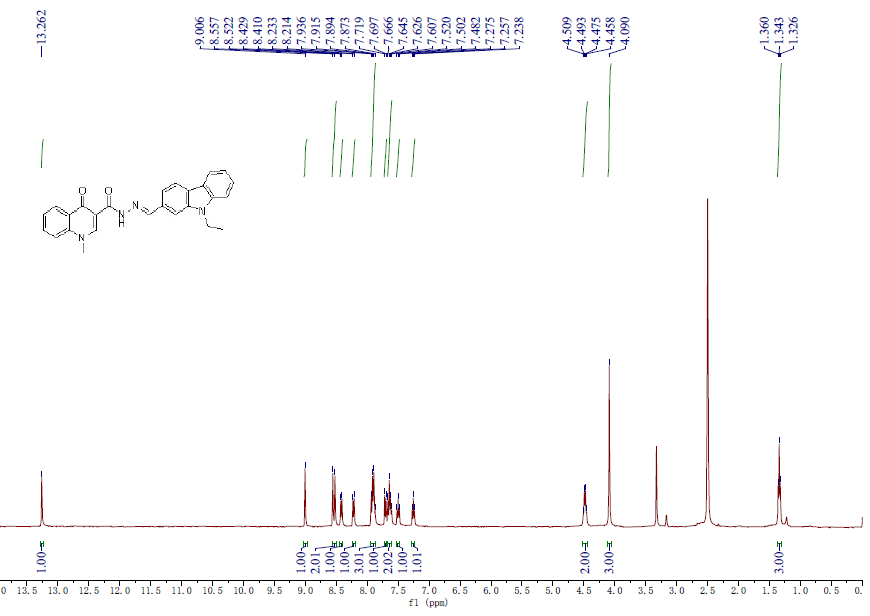


**Fig. S51** ^1^H NMR spectrum of **25**


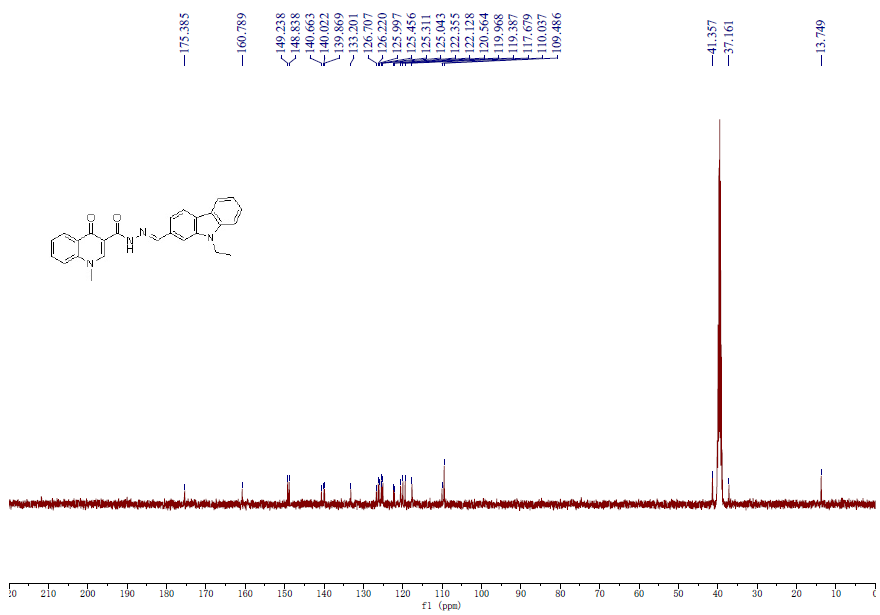


**Fig. S52** ^13^C NMR spectrum of **25**


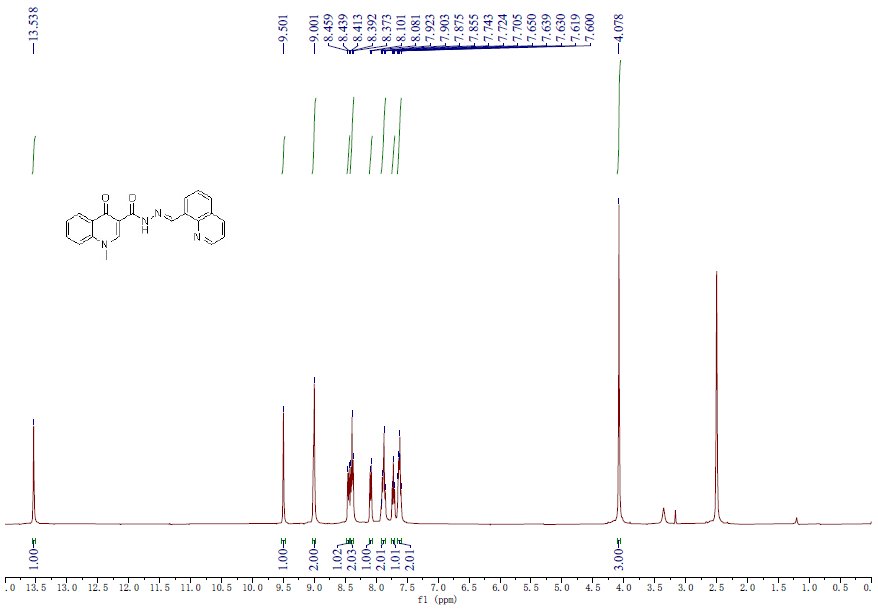


**Fig. S53** ^1^H NMR spectrum of **26**


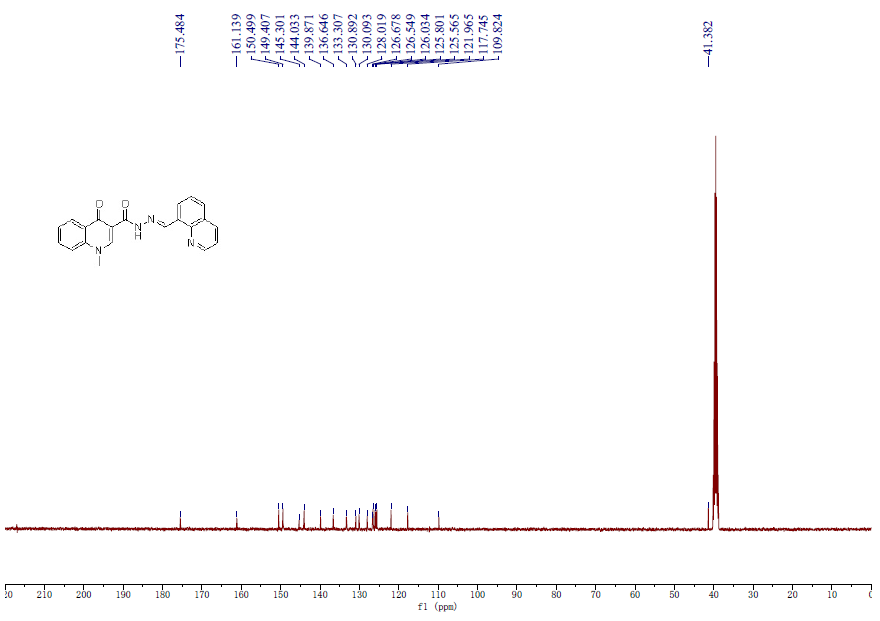


**Fig. S54** ^13^C NMR spectrum of **26**


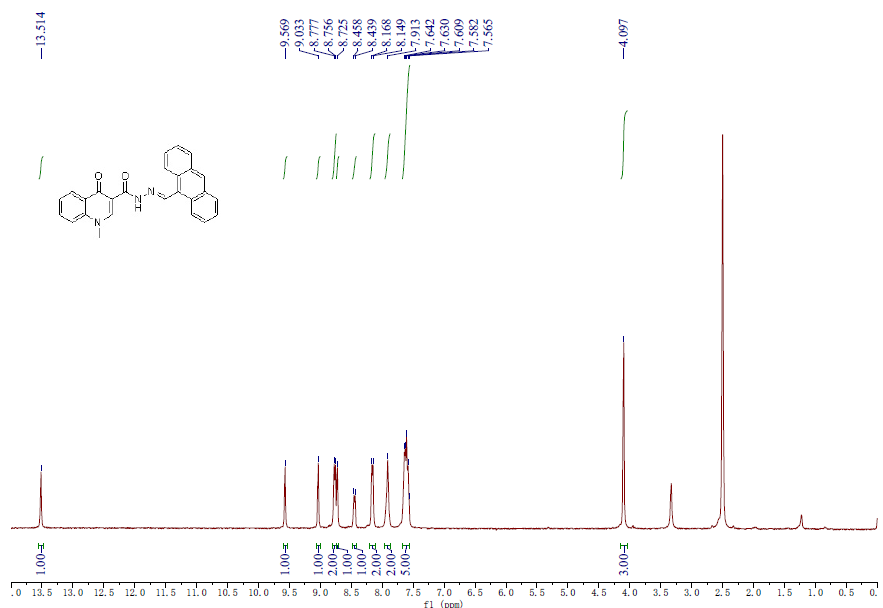


**Fig. S55** ^1^H NMR spectrum of **27**


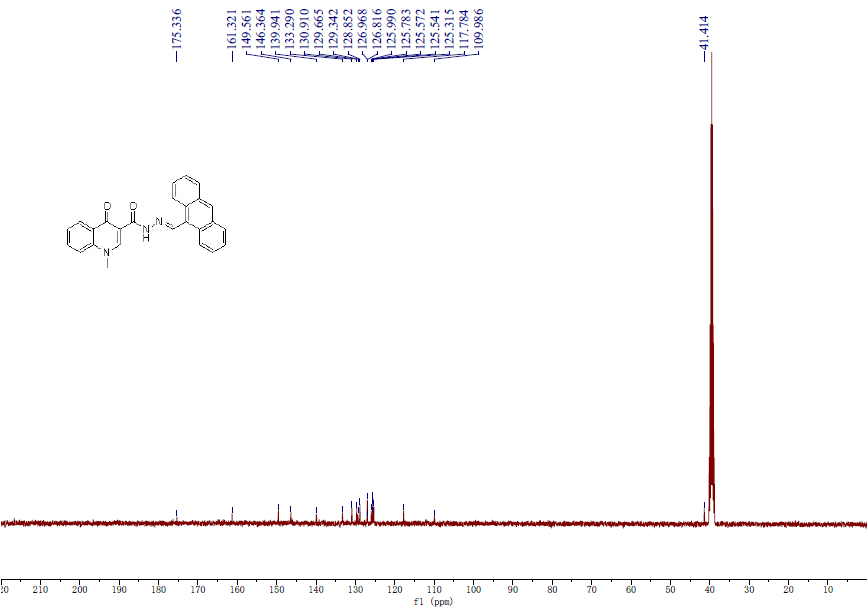


**Fig. S56** ^13^C NMR spectrum of **27**

**Biological assay：**

***Detailed bioassay procedures for the anti-TMV activities.*** Compound solution preparation: Test compound was dissolved in a suitable amount of N, N-dimethyl formate and the solution was diluted to 500 mg/L with water containing 0.1 % TW - 80. The aqueous solution can also be diluted to 100 mg/L.

***Phytotoxic Activity.***The phytotoxic activity test was carried out in a climate chamber at a temperature of 25 °C. The growing 3 - 5 leaf stage tobaccos (*Nicotiana tabacum var Xanthi nc*) were selected. The compound solution (100 mg/L or 500 mg/L) was sprayed on the leaves and the plant height and weight changes were tested after 0, 3, 7 and 10 days respectively. There are threereplicates for each compound.

***Inactivation Effect of Compounds against TMV in Vivo.*** The virus was inhibited by mixing with the compound solution at the same volume for 30 min. The mixture was then inoculated on the growing leaves of the same ages, whereas another pot was inoculated with the mixture of solvent and the virus for control. The local lesion numbers were recorded 3 - 4 days after inoculation. There were three replicates for each compound.

***Curative Effect of Compounds against TMV in Vivo****.* TMV (concentration of 6.0 mg/L) was inoculated on the growing leaves of *N. tabacum L.* of the same ages. Then, the leaves were washed with water and dried. The compound solution was smeared on the leaves, whereas another pot was smeared with solvent for control. The local lesion numbers were then counted and recorded 3 - 4 days after inoculation. There were three replicates for each compound.

***Protective Effect of Compounds against TMV in Vivo.*** The compound solution was smeared on the growing *N. tabacum L.* leaves of the same ages. Another pot was smeared with solvent for control. After 12 h, the leaves were inoculated by the juice-leaf rubbing method and then washed with water. The local lesion numbers appearing 3 - 4 days after inoculation were counted. There were three replicates for each compound.

The juice-leaf rubbing method: Sprinkle emery (500 mesh) on the leaf surface, dip in the virus liquid with a brush, and rub the whole leaf surface along the branch vein twice. Support the underside of the leaf with the palm of the hand. The virus concentration is 10 mg/L. After inoculation, rinse with running water.

The in vitro and in vivo inhibition rates of the compound were then calculated according to the following formula (“av” means average, and controls were not treated with compound):

*inhibition rate (%) = [(av local lesion number of control − av local lesion number of drug treated) / av local lesion number of control] × 100%.*

***Detailed bioassay procedures for the insecticidal activities.***

Larvicidal Activities against diamondback moth (*Plutella xylostella*), cotton bollworm (*Helicoverpa armigera*), corn borer (*Ostrinia nubilalis*) and oriental armyworm (*Mythimna separata*): Stock solutions of each test compound was prepared in dimethylformamide at a concentration of 600 mg/L and then diluted to the required concentration (200, 100, 50, 25, 10, 5, 2, 1 and 0.1 mg/L) with water containing TW - 20. Leaf-dip method was used. Leaf discs (5 cm × 3 cm) were cut from fresh cabbage leaves (or other leaves) and then dipped into the test solution for 3 s. After air-drying, the treated leaf discswere placed individually into vertical tube (or Petri dishes) and the discs were infested with 10 larvae (for example: 10 second-instar diamondback moth larvae, 10 fourth-instar orientalarmyworm larvae). Percentage mortalities were evaluated 3 days after treatment. Evaluations were based on a percentage scale of 0 - 100, where 0 equals no activity and 100 equals total kill. Each treatment was performed three times.

***Larvicidal Activities against Fall Armyworm (Spodoptera Frugiperda (J. E. Smmith)).***

Stock solutions of each test compound was prepared in dimethylformamide at a concentration of 600 mg/L and then diluted to the required concentration (200, 100, 50, 25, 10, 5, 2, 1 and 0.1 mg/L) with water containing TW - 20. Leaf-dip method was used. The leaves of young maize at big bell mouth stage were cut into 5 cm leaf segments, then dipped into the test solution for 10 s, impregnated with drug solution for 10 s. And after air-drying, the treated leaf discs were placed in glass Petri dishes (diameter 75 mm). The well-developed 2nd instar larvae were starved for 4 hours and then connected to the treated leaves. Acetone solvent was used as control. Each treatment of 10 larvae was performed four times. Percentage mortalities were evaluated 3 days after treatment. Evaluations were based on a percentage scale of 0 - 100, where 0 is no activity and 100 is total kill.

***Detailed bioassay procedures for the fungicidal activities.***The compounds were evaluated in mycelial growth tests in artificial media against 14 plantpathogens at rate of 50 mg/L. Test compound was dissolved in a suitable amount of acetone and diluted with water containing 0.1 % TW - 80 to the concentration of 500 mg/L. To each petri dish was added 1 mL such solution and 9 mL culture medium to make a 50 mg/L of medicated tablet, whereas to another petri dish was added 1 mL sterilized water and 9 mL culture medium as blank control. A diameter of 4 mm of hyphae was cut by a hole puncher along the hyphae for bacteria to the outerplate and moved to the medicated tablet. Each treatment was performed three times. The dishes were stored in controlled environment cabinets (24 ± 1 °C) for 48 h, after which the diameter of mycelia growth was investigated and percentage inhibition was calculated.

*Percentage inhibition (%) = (averaged diameter of mycelia in blank controls – averageddiameter of mycelia in medicated tablets) / averaged diameter of mycelia in blank controls*

Larvicidal activities against mosquito (Culex pipiens pal-

lens). Twenty fourth-instar mosquito larvae were placed into the

test solution (10, 5, 2, 1, 0.5, 0.25 and 0.1 mg L

−1

). Percentage mor-

talities were evaluated 8 days after treatment. Evaluations were

based on a percentage scale of 0–100, where 0 is no activity and

100 is total kill. Each treatment was performed three times
